# Influence of Texture and Colour in Breast TMA Classification

**DOI:** 10.1371/journal.pone.0141556

**Published:** 2015-10-29

**Authors:** M. Milagro Fernández-Carrobles, Gloria Bueno, Oscar Déniz, Jesús Salido, Marcial García-Rojo, Lucía González-López

**Affiliations:** 1 VISILAB, Universidad de Castilla-La Mancha, Ciudad Real, Spain; 2 Department of Pathology, Hospital de Jerez de la Frontera, Jerez de la Frontera, Cádiz, Spain; 3 Department of Pathology, Hospital General Universitario de Ciudad Real, Ciudad Real, Spain; Aristotle University of Thessaloniki, GREECE

## Abstract

Breast cancer diagnosis is still done by observation of biopsies under the microscope. The development of automated methods for breast TMA classification would reduce diagnostic time. This paper is a step towards the solution for this problem and shows a complete study of breast TMA classification based on colour models and texture descriptors. The TMA images were divided into four classes: i) benign stromal tissue with cellularity, ii) adipose tissue, iii) benign and benign anomalous structures, and iv) ductal and lobular carcinomas. A relevant set of features was obtained on eight different colour models from first and second order Haralick statistical descriptors obtained from the intensity image, Fourier, Wavelets, Multiresolution Gabor, M-LBP and textons descriptors. Furthermore, four types of classification experiments were performed using six different classifiers: (1) classification per colour model individually, (2) classification by combination of colour models, (3) classification by combination of colour models and descriptors, and (4) classification by combination of colour models and descriptors with a previous feature set reduction. The best result shows an average of 99.05% accuracy and 98.34% positive predictive value. These results have been obtained by means of a bagging tree classifier with combination of six colour models and the use of 1719 non-correlated (correlation threshold of 97%) textural features based on Statistical, M-LBP, Gabor and Spatial textons descriptors.

## Introduction

**Fig 1:**
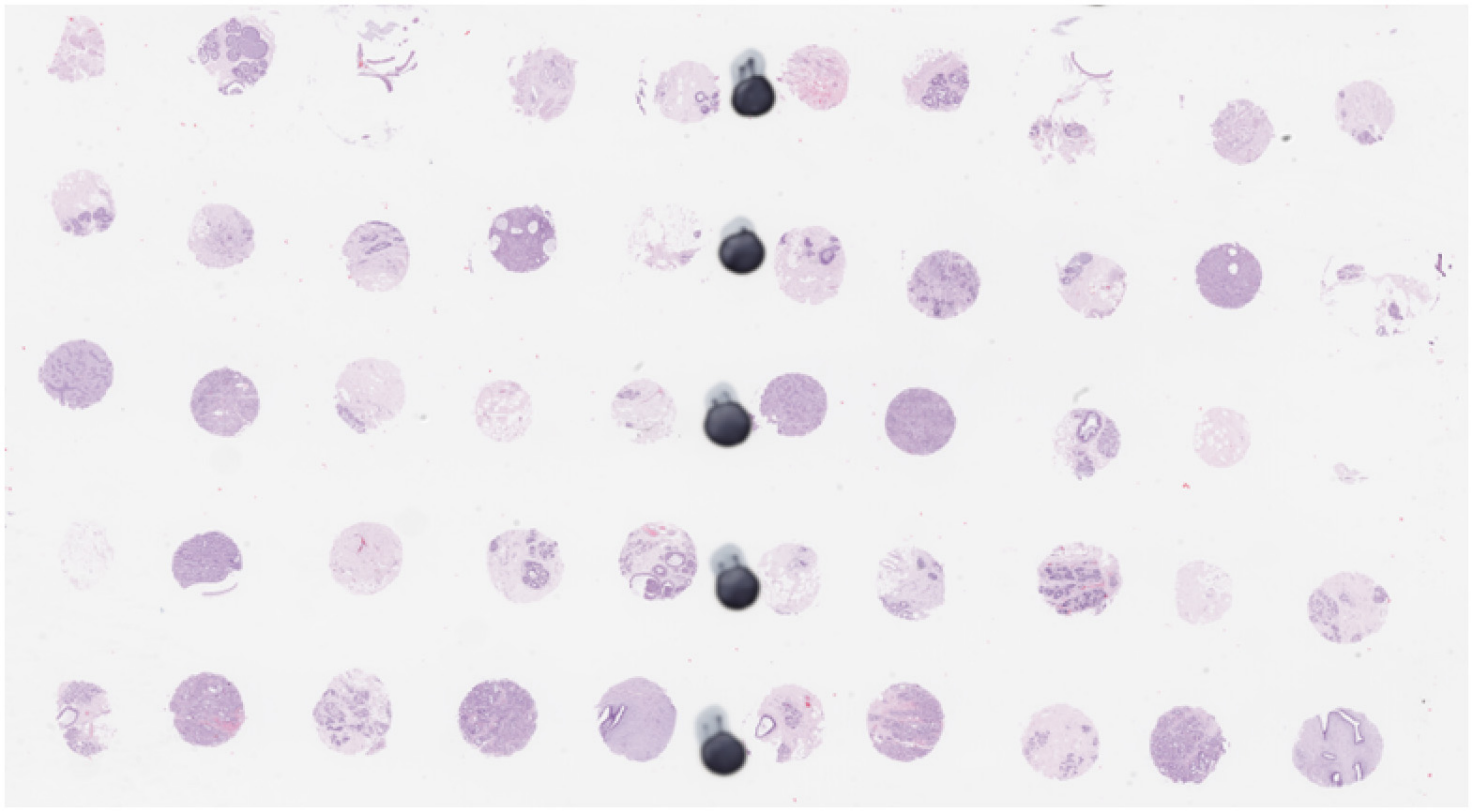
Breast TMA.

The tissue microarray (TMA) is an ordered array that contains several hundreds of small tissue cylinders (core sections) in a paraffin block. A typical example of a breast TMA thumbnail obtained with an Aperio ScanScope T2 is shown in Fig 1. The resolution of Aperio ScanScope T2 at 40x objective is 0.23 *μ*m/pixel. Thus, these cores are images at 40x magnification and their size varies between 6200 and 7300 pixels. These core sections can be cut and processed like any other histological section, using immunohistochemistry (IHC) for protein targets and in situ hybridisation to detect gene expressions or chromosomal alterations [1] [2]. Moreover, TMAs allow rapid and reproducible investigations of biomarkers that define the presence of cancer. However, the use of TMA generates large amounts of information, which requires careful analysis. Currently, this analysis is performed manually under the microscope, which besides being a tedious job that hinders the pathologist workflow, is prone to errors due to subjective interpretations [3].

**Fig 2:**
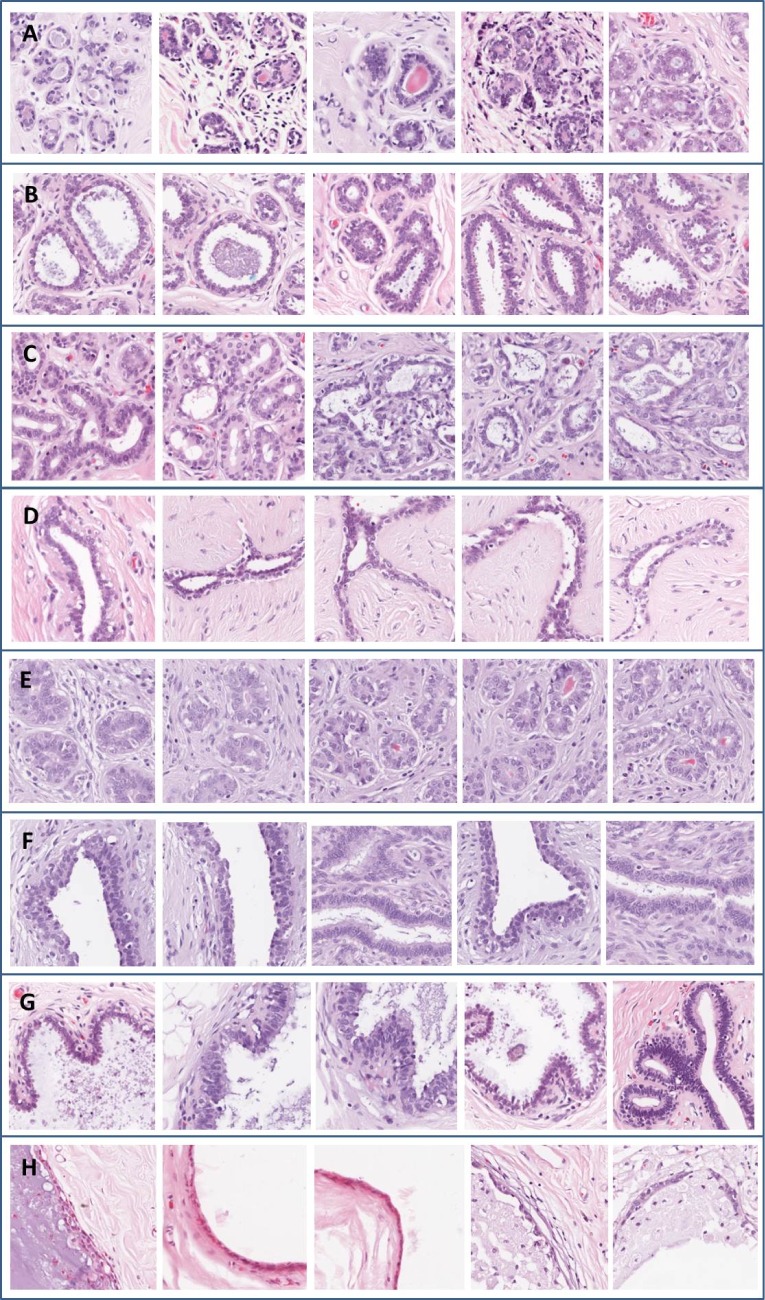
Benign structures and benign anomalous structures in TMA images stained with HE. A) Terminal ducts and lobules, B) Sclerosing lesions (radial scar), C) Adenosis lesions, D) Fibroadenomas, E) Tubular adenomas, F) Phyllodes tumors, G) Columnar cell lesions and F) Duct ectasia.

The first step in the assessment of breast TMA sections by pathologists is classification into different classes, mainly: i) benign stromal tissue with some of cellularity, ii) adipose tissue, iii) benign structures (terminal ducts and lobules) and benign anomalous structures (sclerosing and adenosis lesions, fibroadenomas, tubular adenomas, phyllodes tumours, columnar cell lesions and duct ectasia), and iv) ductal and lobular carcinomas (in-situ or invasive)(see Fig 2). Each core subjected to nuclear staining is then assigned a Quickscore [4] that reflects its immunopositivity. Applying this procedure to breast TMA sections for large numbers of individuals is time consuming and is subject to subjectivity (inter- and intra-observer variability) and misinterpretations [5–7]. Automatic analysis of TMA data is still a challenge due to the broad type of morphologies and stains that can appear in breast tissue structures.

Thus, automatic methods for quantitative analysis and classification of breast TMA image data and, therefore, core diagnosis are desired. The present study is focused on this problem and is tailored to the development of a CAD (computer-aided diagnosis) system in pathology for breast TMA diagnosis.

CAD systems are widely used in radiology, for example, with mammography images to identify and classify lesions [8, 9]. However, CAD systems in histopathology are still a challenge because histopathological images encompass the majority of cancer types. This has led to an increase in the number of studies about methods to distinguish the kinds of malignant tissue. Furthermore, as was mentioned by Gurcan et al. [10], CAD in histopathology is essential not only for disease detection but also to detect obviously benign areas. Pathologists have to analyse daily hundreds of pathologic images, most of them are benign. Therefore, developing a CAD system that classifies benign and malignant tissue in histopathological images is essential to improve the pathologist work. Gurcan et al. also explained that the aim of the CAD system depends on the image scale to use. For instance, the objective in low scales is to capture the tissue architecture, for that, colour or texture methods are commonly used. On the other hand, medium and high scales are more suitable to handle individual histological structures, such as cells, nuclei or glands.

A pattern recognition approach is adopted in this study to model the breast TMA core diagnosis problem. Pattern recognition mainly involves feature extraction and multivariate pattern classification. The former consists in the definition of a set of quantitative measurements, called features or descriptors, defining key attributes of the pattern or region of interest (ROI) to be identified and classified within the image. Therefore, the ROI is described by a point in the multivariate space. Afterwards, a multivariate classifier is then used to define decision boundaries between categories in the input feature space.

Several feature descriptors have been proposed in automated classification of histopathological images, each focus on a different application or ROI. Descriptors may be categorised according to their formulation (see Table 1). Thus, they are broadly divided into six categories: morphological, geometrical, statistical, model-based, signal-processing (space-frequential) and colour model-based. Categories 3 to 5 belong to textural analysis methods.

**Table 1:** Types of models and descriptors: morphological, textural and by colour.

Model	Type of descriptor
Morphological	Shape, roundness, area, perimeter
Geometrical	Voronoi Diagrams
	Structural
Statistical	Co-occurrence matrix (GLCM)—1^*st*^, 2^*nd*^ order
	GL run-length matrices (GLRLMS)
	Higher order—Moments
Model-Based	Markov random fields
	Fractals
Space-Frequential	Fourier
	Wavelets, Gabor
Transformed Space	Textons
	LBP (Local Binary Patterns)
	SIFT (Scale-invariant feature transform)
	HOG (Histogram of oriented gradients)
Colour	RGB, HSV, Lab, CIE-XYZ, Luv

Biomedical specimens do not only exhibit textural information but also colour. Research on the human visual system suggests that the image signal is composed of a luminance and a chrominance component. In the human eye, chrominance is processed at a lower spatial frequency than luminance. Furthermore, it has been shown that much of the discriminative texture information is contained in high spatial frequencies. It seems that texture information is associated with the luminance component, whereas chrominance is associated with homogeneous regions [11]. Therefore, colour is also an important feature, and its interrelationship with texture should be considered when possible [12].

Table 2 summarises different state-of-the-art feature descriptors used in histopathology, the classification methods and their accuracy. The number of classes has also been included, as well as the properties of the database (BBDD). These properties include the number of images used in the classification process, if they are whole slide images (WSI) or not, the acquisition scale and the device used in the acquisition process.

**Table 2:** Classification performance of different state-of-the-art methods in digital pathology.

Author	Feature Descriptor	Classifier	Num. Classes	BBDD Properties	Results
**Breast TMA**					
Yang [45]	Textons	Adaboost	2	300 WSI, 45 TMAs, Trestle MedMicro, 40x, RGB	89.00% ACC
Qi [46]	LBP, 2^*nd*^ order statistics	AdaBoost with Linear perceptron least-square	2	92 TMAs, 10x, RGB, Multispectral	88% accuracy
Amaral [5]	Gaussian filters	NN	4	344 cores, RGB	75.00% ACC
Le [7]	Quadrature mirror filter (QMF)	SVM	4	520 cores, RGB	80.42% ACC
Xing [6]	Textons	Adaboost	4	547 ROIs, RGB	88.00% ACC
Fernández-Carrobles [12]	Textons	AdaBoost, Bagging Trees	4	628 ROIs, 10x, Aperio ScanScope RGB, CMYK, HSV, Lab, Luv SCT, Hb, Lb	98.1% ACC
Proposed Method	Fourier, Wavelets, Gabor, M-LBP, Textons	Fisher, SVM Random Forest, Bagging Trees, AdaBoost	4	628 ROIs, 10x, Aperio ScanScope ALIAS II RGB, CMYK, HSV, Lab, Luv SCT, Hb, Lb	99.05% ACC
**Renal TMA**					
Fuchs [47]	LBP	Random Forest	2	133 cores, Nanozoomer C9600 40x, RGB	0.026 p-value
**General TMA**					
Ahonen [48]	LBP	SVM	2	1296 ROIs, Mirax Scan, 20x, RGB	99.5% ACC
**Breast Biopsy**					
Niwas [15]	logGabor	SVM	2	610 ROIs, Aperio ScanScope, 20x, HSI	98.6% ACC
Chekkoury [49]	Textons	SVM	2	100 ROIs, 40x, CMY	87.00% ACC
Zhang [16]	CLBP, 2^*nd*^ order statistics, Curvelet transform	SVM, Multi-Layer Perceptron	3	361 ROIs, Nikon Eclipse E600, 40x, RGB	99.25% ACC
Bahlmann [50]	1^*st*^ order statistics	SVM	2	DMetrix, 40x, RGB	98.6% ACC
**Prostate Biopsy**					
Farjam [51]	Roundness, shape, Haralick Wavelets	Linear	5	290 ROIs, RGB	90% ACC
Doyle [52]	Haralick, Gabor	AdaBoost Cascade	2	22 ROIs (3 scales), 40x, HSV	88% ACC
Doyle [53]	Architectural, Morphological, Haralick, Gabor	SVM	4	54 ROIs, 40x, RGB	89.36% ACC
Huang [54]	Multiwavelet, Gabor, 2^*st*^ order statistics, Fractals	Bayesian, K-NN, SVM	5	205 ROIs, RGB	94.7% ACC
Khurd [55]	Textons	SVM	2	75 ROIs, 10x, RGB	93.70% ACC
Monaco [56]	Area, homogeneity size	Probabilistic pairwise Markov models	2	40 WSI, Aperio, 10x, Lab	87% sensitivity
Xu [57]	Diffeomorphic filters	SVM	4	23 WSI, 105 images, Aperio, 20x, RGB, HSV	82.5% ACC
DiFranco [22]	2^*st*^ order statistics	Random Forest, SVM	2	15 WSI, Aperio XT 40x, RGB, Lab	95% AUC
Doyle [58]	Haralick Gabor	Boosted Bayesian	2	100 WSI, Aperio ScanScope 40x, HSI	81% AUC
**Head an Neck Biopsies**					
Mete [59]	Clustering	SVM	2	7 WSI, 20x, RGB	96% ACC
**Brain Biopsy**					
Lessmann [60]	colour transforms, Wavelets	Self Organizing Map	4	1280 ROIs, Zeiss Axioskop 2 Plus, RGB	79% ACC
**Neuroblastoma on biopsy**					
Kong [61]	Haralick,	KNN, SVM, Bayesian LDA	3	33 WSI Aperio ScanScope T2 40x, RGB, Lab	87.88% ACC
**Mitotic Cells—Breast CAD**					
Nateghi [62]	Haralick, GLRLMS, moments, CLBP, Wavelets, Gabor	SVM	2	35 WSI, Aperio XT, 40x, RGB	77.34% F-measure
Tashk [63]	LBP	SVM	2	5 WSI, Aperio XT, Hamamatsu 40x, RGB	70% F-measure

The research literature using a combination of colour and texture for features description is limited and has not been significantly explored in digital pathology. The research shows that no standard colour model is used. Moreover, there is also a lack of comprehensive studies of the most suitable colour models for the different tissues types in histopathology [12–14].

Other problems are that the database is poorly populated and the number of classes to classify is insufficient. Though some promising results have been shown for breast biopsy classification with 98.6% and 99.25% accuracy [15, 16], these works use only two classes. In breast tissue there are other structures that must be considered, such as, adipose tissue and benign anomalous structures.

This study presents an exhaustive assessment on the utility of texture and colour models never considered before for automatic classification of breast TMA into four classes or structures. To this end, four different texture models comprising six types of descriptors and eight colour spaces were analysed in order to find which descriptor or combination of descriptors best discriminate between breast TMA structures. We found significant performance differences among descriptors and a significant improvement when certain groups of descriptors are combined with different colour models, providing an overall accuracy rate of 99.88%.

The remainder of the paper is organised as follows: Section 2 presents the material and methods used in this work. Materials include the eight colour models applied on our histopathological images and the hardware employed to perform the experiments. In Section 3, the feature descriptors, the feature extraction process and the feature dimension reduction methods are described. Section 4 addresses the classification process including an explanation of the classifiers used. Section 6 presents the experimental results divided into four different experiments that combine colour models and descriptors. Finally, Section 7 concludes the paper.

## Materials and Methods

This study was reviewed and approved by the ethics committee at Hospital General Universitario de Ciudad Real. This is a retrospective cohort study and no identifying information was taken from the patients. We used only the digital image obtained from the scanner at the Hospital General Universitario de Ciudad Real. The TMA images were acquired by the motorised microscope ALIAS II (LifeSpan Biosciences Inc.) and by an Aperio ScanScope T2 at 10x. Once the breast TMA cores were digitalised, 628 representative regions of the four tissue classes were selected. These regions of interest were selected in a manual way and under the supervision of a pathologist. The size of these regions was 200 x 200 pixels (0.74*μ*m/pixel at 10x) and the TMA tissue classes were: i) benign stromal tissue with low and medium cellularity (170 images), ii) adipose tissue (103 images), iii) benign structures and anomalous (163 images), and iv) different kinds of malignancy, that is, ductal and lobular carcinomas (192 images). The first class (i) is characterised by the pink hue-blue stromal cells prior staining due to tissue with HE (hematoxylin and eosin). The second class (ii) is represented in the images as bubbles on the tissue stroma. The third class (iii) shows lobules, ducts and several anomalous structures. The types of anomalous benignity represented in class three are: sclerosing and adenosis lesions, fibroadenomas, tubular adenomas, phyllodes tumours, columnar cell lesions and duct ectasia (see Fig 2). Finally, the fourth class (iv) is characterised by the different kinds of malignancy. Images of this class show ductal and lobular carcinomas in situ and invasive.

Experiments were performed on an Intel Core i7 950 3.07 GHZ computer with 12 GB RAM. The method was implemented using C/C++ and the IPP and OpenCV libraries for image processing. The Intel TBB library was used for parallelisation of the algorithms. Classifiers used in this paper were extracted from the PRTools (Pattern Recognition Tools) MATLAB toolbox.

## Feature Descriptors and Feature Extraction

### Colour Model Analysis

In order to better represent the HE images and the colour patterns that the human visual system perceives, we used six colour models: RGB, CMYK, HSV, Lab, Luv, SCT and two combinations of them Lb and Hb. The eight colour representations were compared individually and jointly. In this way we addressed two issues: (i) characterisation of the HE images with limited colour spectrum, i.e., only with blue and pink hues; (ii) representation of the three components that the human visual system perceives, i.e., luminance, chrominance and an achromatic pattern component hence, the importance of analysing colour models in histopathological analysis. The eight colour spaces are described as follows:

- **RGB**. In the RGB colour model, each colour appears as a combination of the three primary spectral components: red, green, and blue. The RGB model is based on a three-dimensional Cartesian coordinate system. This is represented by a cube with the corners corresponding to the red, green, blue, cyan, magenta, yellow, black (0,0,0) and white (255,255,255) colours. Grey scale extends in a diagonal from black to white corners of the cube. Colours are the points on or inside the cube, defined by vectors extending from the origin.
- **CMYK**. The CMYK colour model is a subtractive colour model contrary to the RGB which is an additive colour model. This colour model refers to the four inks used in colour printing: cyan, magenta, yellow, and key (black). In subtractive colour models black colour is the absence of light and other colours are the combination of all primary coloured lights. This colour model has been used in studies about tissues biomarkers by IHC for cervical cancer [17] and intra-epithelial lesions [18].
- **HSV**. The HSV model is a nonlinear transformation of the RGB colour space that describes colour (hue) in terms of their shade (saturation) and brightness (value). This is represented by a cone. Hue (base cone) is expressed as a number from 0 to 360 degrees representing hues of red (starts at 0), yellow (starts at 60), green (starts at 120), cyan (starts at 180), blue (starts at 240), and magenta (starts at 300). Saturation (radius base) is the amount of gray (0% to 100%) in the colour. Finally, value (cone height) works in conjunction with saturation and describes the brightness or intensity of the colour from 0% (black) to 100% (white). HSV and HSI have also been used in several studies with histological images of prostate [19] or breast stained with diaminobenzidine and hematoxylin (DABH) [20].
- **Lab**. The Lab colour model (also called CIE L*a*b* colour model) was specified by The International Commission on Illumination (CIE). This was designed to approximate human vision and is composed of three colour channels: L, a and b. The L channel indicates colour luminosity, black and white take values 0 and 100, respectively. The *a* channel indicates the colour position between magenta and green (negative values indicate green while positive values indicate magenta). Lastly, the *b* channel indicates the colour position between yellow and blue (negative values indicate blue while positive values indicate yellow). This colour model has been used in histological images due their capability to segment the different tissue structures, including stroma, cells and lumen [19, 21, 22].
- **Luv**. Luv (also called CIE L*u*v* colour model) is a colour model adopted by the CIE in 1976 as a modification of U*V*W* and Lab colour models. The Luv model is especially useful when working with a single illuminant and uniform chrominance. The L channel represents colour luminosity as in Lab. The chromaticity components u and v are coordinates of a specified white point, a white point being a set of values or chromaticity coordinates that serve to define the neutral colour in an image. Therefore, the Luv colour model has been used for illumination normalisation [10, 16].
- **SCT**. The SCT colour model (spherical coordinate transform) is not a colour model per se. Nevertheless, it is possible to make a conversion to SCT based on the RGB colour model. Thus, the SCT colour model is decomposed into three components M, *ϕ* and *θ*. M reproduces the colour intensity and is the length of the RGB vector, *ϕ* is the angle between the blue axis to the RG plane and *θ* corresponds to the angle between G and R axes [23]. Conversion from RGB to SCT is shown in Eqs (1), (2) and (3). This colour model is useful when there are few changes in illumination.
  $$\begin{array}{c} M=\sqrt{R^{2}+G^{2}+B^{2}} \end{array}$$ (1)
  $$\begin{array}{c} \phi =cos^{-1}\left(\frac{B}{M}\right) \end{array}$$ (2)
  $$\begin{array}{c} \theta =tan^{-1}\left(\frac{G}{R}\right) \end{array}$$ (3)
- **Lb and Hb**. The Lb and Hb colour spaces consist of the L, H and b colour channels. The emphasis on the L and b channel (present in Lab and Luv colour models) is due to the ability of these channels to make a good segmentation on breast TMA cells. The use of L and b channels has shown to be essential to improve classification results. Images representing Lb and Hb are visualized by duplicating the b channel; thus, the three colour components are Lbb and Hbb.

**Fig 3:**
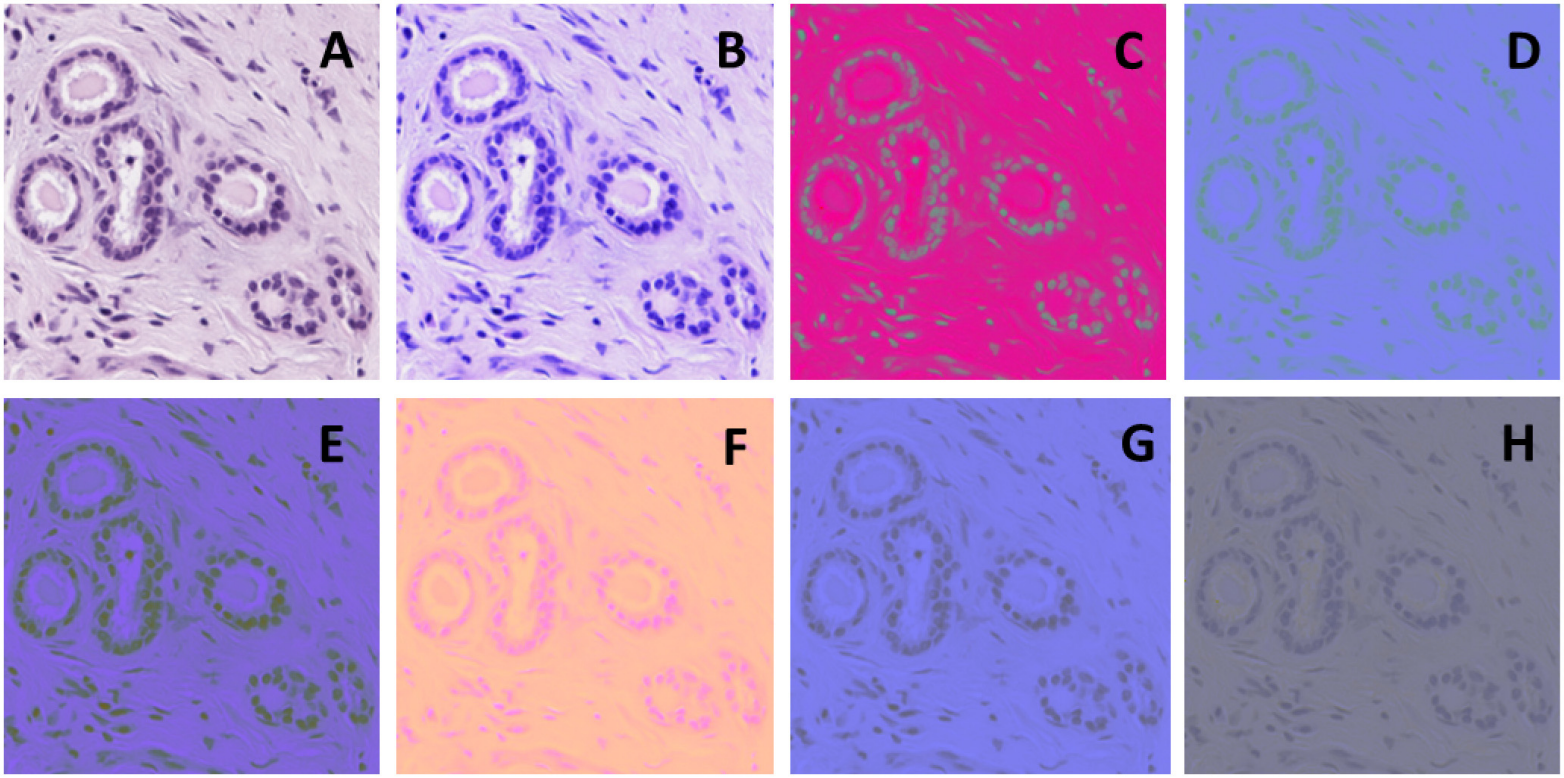
Colour models and combinations. A) RGB, B) CMYK, C) HSV, D) Lab, E) Luv, F) SCT, G) Lbb, H) Hbb.

TMA image samples with the eight colour spaces are shown in Fig 3.

#### Colour model combination

Surprisingly, only one paper has studied the influence of colour models in HE histological images [24]. A combination of colour models is one of the goals of this paper. Usually, all the studies about tissue classification use only the most simple colour model, which is the RGB. Furthermore, channels and colour models of each colour model can be combined to create new models. Our study not only performs a classification using the aforementioned eight colour models individually, but also carries out the classification making combinations of them. We found that the classification results improved when more than two colour models were combined.

Textural features will be one of the bases of this study. We organised descriptors in groups according to their formulation, as shown in Table 1, which at the same time will help to conduct the later experiments. A brief description is given here and we refer the reader to plenty of well documented references.

### Statistical Descriptors

In 1973, Haralick introduced a general procedure for describing textural features of an image [25]. First and second order statistical descriptors are a quantification of the spatial variation in the spatial image shade. The first order statistical descriptors are based on the image histogram (Table 3). On the other hand, the second order statistical descriptors consider the relationship of the image pixels (see Table 4). They are based on the Grey Level Co-occurrence Matrix (GLCM) of the image. GLCM are second order histograms that represent the spatial dependence of the image pixels. These spatial relationships are calculated with the neighbouring pixels in a sliding window. Furthermore, these relationships can be defined indicating the distance and the angle between the reference pixel and its neighbour. Distances were taken at 1, 3 and 5 pixel-wide neighbourhoods and at a direction parameter equal to 0°, 45°, 90° and 135° to cover different angles. Finally, a total of 241 texture features were extracted by the first and second order statistical descriptors, 13 and 19 features respectively, as suggested in [12].

**Table 3:** 1^*st*^ order statistical descriptors.

Statistical	Formula
Mean	$\sum_{i=0}^{N-1}ih(i)$
Mode	*i*∣*h*(*i*) = *max*(*h*)
Variance	$\sum_{n=0}^{N-1}{\left(i-\mu \right)}^{2}h(i)$
1^*er*^ quartile	$\frac{N}{4}$, if N is pair
	$\frac{N+1}{4}$, if N is odd
2^*o*^ quartile	$\frac{2N}{4}$, if N is pair
	$\frac{2N+1}{4}$, if N is odd
3^*er*^ quartile	$\frac{3N}{4}$, if N is pair
	$\frac{3N+1}{4}$, if N is odd
Interquartile range	3^*er*^ quartile—1^*er*^ quartile
Minimum	*Min*(*h*(*i*))
Maximum	*Max*(*h*(*i*))
Range	*Max*(*h*(*i*)) − *Min*(*h*(*i*))
Entropy	$\sum_{i=0}^{N-1}h(i)log(h(i))$
Asymmetry	$\frac{1}{\sigma^{3}}\sum_{n=0}^{N-1}{\left(i-\mu \right)}^{3}h(i)$
Kurtosis	$\frac{1}{\sigma^{4}}\sum_{n=0}^{N-1}{\left(i-\mu \right)}^{4}h(i)$

**Table 4:** 2^*nd*^ order statistical descriptors.

Statistical	Formula
Energy	$\sum_{i=0}^{N-1}\sum_{j=0}^{N-1}p{\left(i,j\right)}^{2}$
Contrast	$\sum_{n=0}^{N-1}n^{2}\left(\sum_{i=0}^{N-1}\sum_{j=0}^{N-1}p(i,j)\right)$, ∣*i* − *j*∣ = *n*
Correlation	$\frac{\sum_{i=0}^{N-1}\sum_{j=0}^{N-1}(ij)p(i,j)-\mu_{x}\mu_{y}}{\sigma_{x}\sigma_{y}}$
Variance	$\sum_{i=0}^{N-1}\sum_{j=0}^{N-1}{\left(i-\mu \right)}^{2}p(i,j)$
Sum average	$\sum_{i=0}^{2(N-1)}ip_{x+y}(i)$
Sum entropy	$\sum_{i=0}^{2(N-1)}p_{x+y}(i)log(p_{x+y}(i,j))$
Sum variance	$-\sum_{i=0}^{2(N-1)}{\left(i-SumEntropy\right)}^{2}p_{x+y}(i)$
Homogeneity 1	$\sum_{i=0}^{N-1}\sum_{j=0}^{N-1}\frac{p(i,j)}{1+{\left(i-j\right)}^{2}}$
Entropy	$-\sum_{i=0}^{N-1}\sum_{j=0}^{N-1}p(i,j)log(p(i,j))$
Difference variance	$\sum_{i=0}^{N-1}i^{2}p_{x-y}(i)$
Difference entropy	$-\sum_{i=0}^{N-1}p_{x-y}(i)log(p_{x-y}(i,j))$
Measure of correlation 1	$\frac{HXY-HXY1}{max(HX,HY)}$
Measure of correlation 2	${\left(1-exp[-2.0(HXY2-HXY)]\right)}^{\frac{1}{2}}$
Homogeneity 2	$\sum_{i=0}^{N-1}\sum_{j=0}^{N-1}\frac{p(i,j)}{1+|i-j|}$
Cluster Shade	$\sum_{i=0}^{N-1}\sum_{j=0}^{N-1}{\left(i+j-\mu_{x}-\mu_{y}\right)}^{3}p(i,j)$
Cluster Prominence	$\sum_{i=0}^{N-1}\sum_{j=0}^{N-1}{\left(i+j-\mu_{x}-\mu_{y}\right)}^{4}p(i,j)$
Autocorrelation	$\sum_{i=0}^{N-1}\sum_{j=0}^{N-1}(ij)p(i,j)$
Dissimilarity	$\sum_{i=0}^{N-1}\sum_{j=0}^{N-1}|i-j|p(i,j)$
Maximum probability	*max*(*p*(*i*, *j*)), *i* = 0 … *N* − 1, *j* = 0 … *N* − 1
When:	
$\mu_{x}=\sum_{i=0}^{N-1}\sum_{j=0}^{N-1}ip(i,j)$ $p_{x}(i)=\sum_{j=0}^{N-1}p(i,j)$	
$\mu_{y}=\sum_{i=0}^{N-1}\sum_{j=0}^{N-1}jp(i,j)$ $p_{y}(j)=\sum_{i=0}^{N-1}p(i,j)$	
$\sigma_{x}=\sqrt{\sum_{i=0}^{N-1}p_{x}(i){\left(i-\mu_{x}\right)}^{2}}$ $\sigma_{y}=\sqrt{\sum_{j=0}^{N-1}p_{y}(i){\left(i-\mu_{y}\right)}^{2}}$	
$p_{x+y}(k)=\sum_{i=0}^{N-1}\sum_{j=0}^{N-1}p(i,j)$, *i* + *j* = *k*, *k* = 0 … 2(*N* − 1)	
$p_{x-y}(k)=\sum_{i=0}^{N-1}\sum_{j=0}^{N-1}p(i,j)$, ∣*i* − *j*∣ = *k*, *k* = 0 … *N* − 1	
$HXY=-\sum_{i=0}^{N-1}\sum_{j=0}^{N-1}p(i,j)log(p(i,j))$	
$HXY1=-\sum_{i=0}^{N-1}\sum_{j=0}^{N-1}p(i,j)log(p_{x}(i)p_{y}(j))$	
$HXY2=\sum_{i=0}^{N-1}\sum_{j=0}^{N1}p_{x}(i)p_{y}(j)log(p_{x}(i)p_{y}(j))$	

### Space-Frequency Descriptors

**Fig 4:**
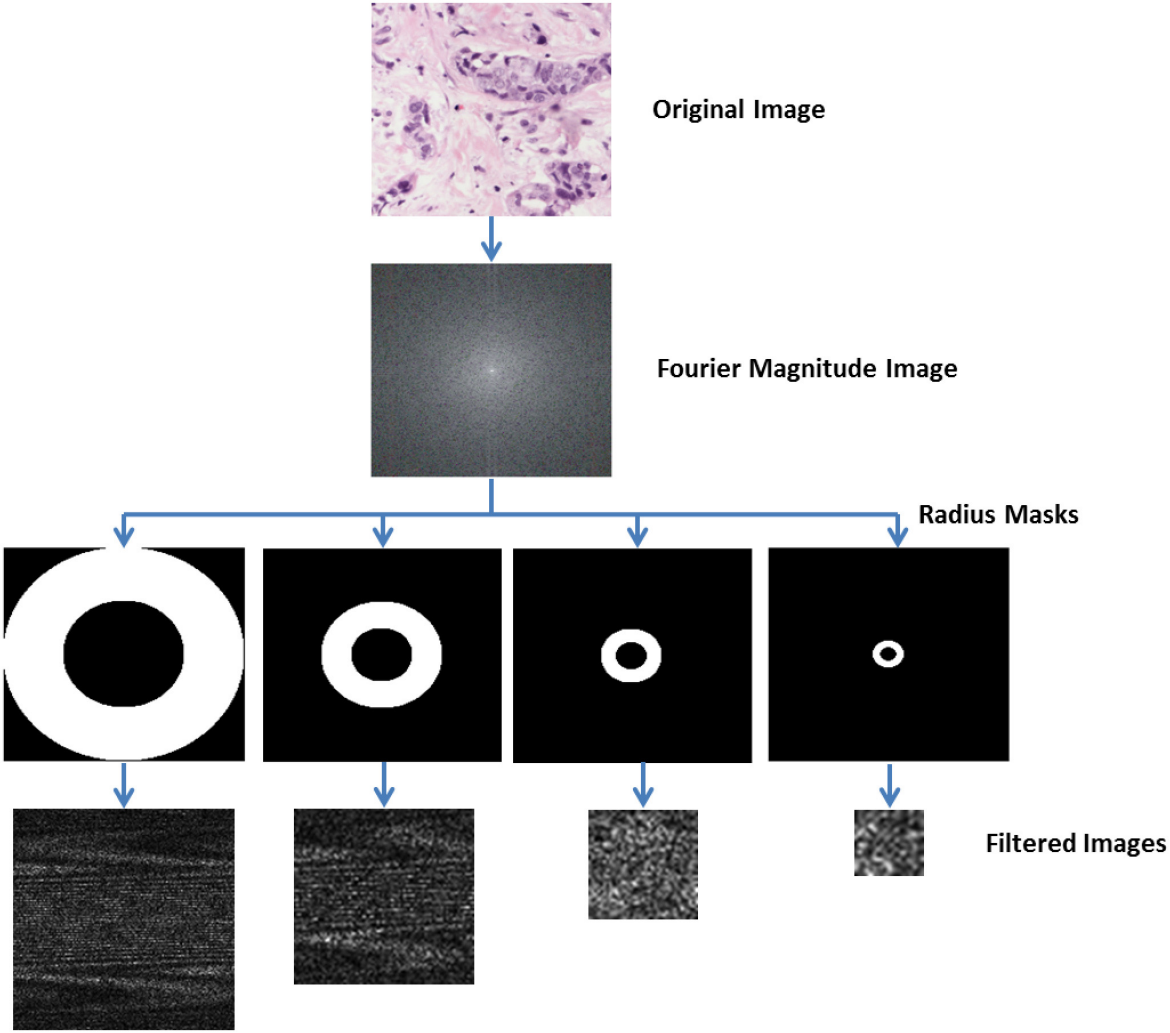
Process to extract the Fourier filtered images.

**Fig 5:**
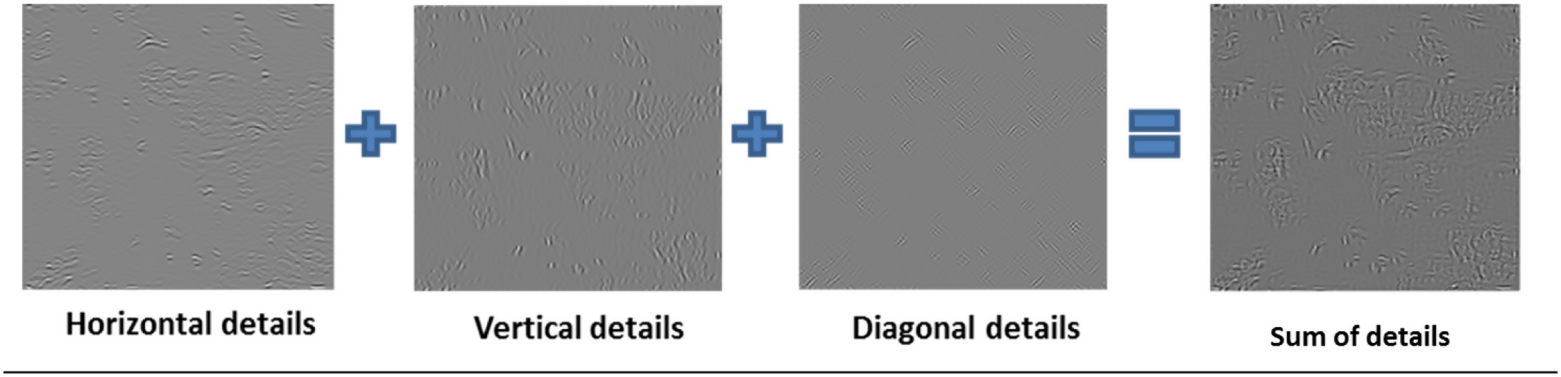
Filtered wavelet images obtained by adding the three detail images.

1. **Fourier Transform**
  The Fourier Transform (FT) is an important image processing tool which is used in a wide range of applications to analyse spectral content of a signal where 2D frequencies arise from grey level variations along features such as, image filtering, image reconstruction and image compression. Fourier is a frequency descriptor. The FT embeds translational, periodicity, rotation and scaling properties. The spectrum of the FT encloses the direction of repeating structures of the image as high concentrations of energy in the spectrum. This allows the extraction of certain parts of the image with much detail.
  In order to define the 2D FT let us suppose that the continuous function *f*(*x*, *y*) has been discretised in the succession: {*f*(*x*_0_, *y*_0_), *f*(*x*_0_ + △*x*, *y*_0_ + △*y*), *f*(*x*_0_ + 2△*x*, *y*_0_ + 2 △*y*), …, *f*(*x*_0_ + [*M* − 1]△*x*, *y*_0_ + [*N* − 1]△*y*)} taking MxN samples. The function *f*(*x*, *y*) can be defined by *f*(*x*, *y*) = *f*(*x*_0_ + *x*△*x*, *y*_0_ + *y*△*y*) where *x* takes the values 0, 1, 2, …, M-1 and *y* takes the values 0, 1, 2, …, N-1. Bearing this in mind, the 2D discrete Fourier transform (DFT) pair, which is composed by the 2D discrete and the 2D discrete inverse Fourier transforms, is defined as in Eqs (4) and (5) [26].
  $$\begin{array}{c} F\left(u,v\right)=\frac{1}{MN}\sum_{x=0}^{M-1}\sum_{y=0}^{N-1}f\left(x,y\right)e^{-j2\pi (ux/M+vy/N)} \end{array}$$ (4)
  for *u* = 0, 1, 2, …, M-1 and *v* = 0, 1, 2, …, N-1.
  $$\begin{array}{c} f\left(x,y\right)=\sum_{u=0}^{M-1}\sum_{v=0}^{N-1}F\left(u,v\right)e^{j2\pi (ux/M+vy/N)} \end{array}$$ (5)
  for *x* = 0, 1, 2, …, M-1 and *y* = 0, 1, 2, …, N-1.
  In an image using the 2D DFT, variables *x* and *y* denote columns and rows, and variables *u* and *v* denote the vertical and horizontal frequencies, respectively. Horizontal and vertical frequencies are represented in the Fourier magnitude image as vertical and horizontal lines, respectively. The central point in the image is the *direct current* term that represents the average intensity of the whole image. Once the 2D DFT is applied on an image it is possible to distinguish the discontinuities of the original image in the Fourier magnitude image.
  For this paper, four filtered images were calculated by image. On the Fourier magnitude image, different frequency radius masks were extracted. This radius represents the different frequencies in the image (see Fig 4). Corresponding pixels of each mask in the magnitude image are placed in a matrix of pixels, which is then converted to an image.
2. **Wavelets Transform**
  Fourier transform has only frequential information. Therefore, it is necessary to use a function that contains frequential and spatial information, like wavelets. In this way, the Wavelet Transform (WT) can be defined as a special type of transform represented by versions of a shifted and scaled finite wave. This wave is denominated *mother wavelet* [27].
  A set of base functions (or base wavelets) *ψ*_*j*, *k*_(*x*) is generated from the wavelet mother *ψ*_*x*_ by the expression defined in Eq (6) where the variable *j* determines the scale and *k* the translations. Thus, the scale j is bigger than 0 and the translations k are real numbers.
  $$\begin{array}{c} \psi_{j,k}=\frac{1}{\sqrt{j}}\psi \left(\frac{x-k}{j}\right) \end{array}$$ (6)
  A wavelet function is an orthonormal wavelet if the set of base functions *ψ*_*j*, *k*_(*x*) defined by the Eq (7) forms orthonormal bases in *L* ^2^(*R*)
  $$\begin{array}{c} \psi_{j,k}\left(x\right)=2^{j/2}\psi \left(2^{j}x-k\right) \end{array}$$ (7)
  As in the case of the DFT, suppose that the continuous function *f*(*x*) is a set of *M* samples: *f*(*x*) = *f*(*t*_0_ + *x*Δ*t*) where *x* = 0, 1, …, M-1 for some initial time (*t*_0_) and sampling period Δ*t*. In the case of the discrete WT (DWT) a scale function (*father wavelet*) is required *ϕ*(*x*). So, the base functions *ϕ* = *ϕ*(0), *ϕ*(1), …, *ϕ*(*M* − 1)^*T*^ and *ψ* = *ψ*(0), *ψ*(1), …, *ψ*(*M* − 1)^*T*^ also contain *M* elements.
  Taking the following values: *j*_0_ = 0, *M* = 2^*J*^, *j* = 0, 1, 2, …, *J* − 1 and *k* = 0, 1, 2, …, 2^*j*^ − 1, the DWT is defined by the basis functions shown in Eqs (8) and (9).
  $$\begin{array}{c} W_{\phi }\left(j_{0},k\right)=<f\left(x\right),\phi_{j_{0},k}>=\frac{1}{\sqrt{M}}\sum_{x=0}^{M-1}f\left(x\right)\phi_{j_{0},k}\left(x\right) \end{array}$$ (8)
  $$\begin{array}{c} W_{\psi }\left(j,k\right)=<f\left(x\right),\psi_{j,k}>=\frac{1}{\sqrt{M}}\sum_{x=0}^{M-1}f\left(x\right)\psi_{j,k}\left(x\right),\;\;\;\;\text{(}j>j_{0}\text{)} \end{array}$$ (9)
  Where *W*_*ϕ*_(*j*_0_, *k*) and *W*_*ψ*_(*j*, *k*) are called *approximation coefficient* and *detail coefficient*, respectively.
  The DWT applied to an image can be described as a low-resolution image at scale *n*, plus a set of details on it ranging from low to high resolution [28]. The 2D DWT is implemented by applying low-pass and high-pass filters to the rows and columns of the image. In this way, the original image is divided into four sub-images: low resolution (scale function), vertical, horizontal and diagonal orientation (tree basis wavelets or details). The low-resolution sub-image is obtained by convolving the low-pass filter on the columns and rows of the image. The vertical oriented sub-image is calculated from the convolution of the low-pass filter on the image columns and the high-pass filter on the image rows. The horizontal orientation image is extracted from the convolution of the high-pass filter and the low-pass on the image columns and rows, respectively. Finally, the diagonal orientation image is obtained from the convolution of the high-pass filters on the image rows and columns. All these images, in the same scale, must be summed to achieve orientation invariance. Based on preliminary observations, the number of scales was set to four and the wavelet used was the overcomplete version and five stem long of Daubechies basis to build our descriptors as the energy on every scaled level.
  Finally, four scaled levels were selected in this paper. Only the detail images of each level were used leaving out the scaled image. Additionally, these detail images were summed to merge the three detail images on a single image (see Fig 5).
3. **Multiresolution Gabor Transform**
  2D Gabor filters are bandpass filters used in image processing for edge detection, segmentation and feature extraction. The Gabor filter consists of a Gaussian function modulated by complex sinusoidal of frequency and orientation. The result is the partition of the Fourier plane into bands modulated in orientation and octave bands apart in frequency. Gaussian shape ensures an optimum spreading in both dimensions, i.e., space location and frequency discrimination, while one weakness of wavelets is the pronounced frequency overlapping. However, the Gabor filter is not suitable for some applications. The DC term has non-zero mean value at some specific bandwidths, being counter-productive in pattern recognition applications. The DC component is not necessary due to the fact that it gives a feature that changes with the average value. Another drawback of the Gabor filters is that filters of arbitrarily wide bandwidth cannot be implemented since the filter width is limited to one octave. A multiresolution analysis for the Gabor filter can solve some of its limitations. The multiresolution analysis transforms the filters on different frequencies as scaled versions of each other [29]. Thus, fine detail is equivalent to high frequency. The multiresolution 2D Gabor filter in the spatial domain with *l* scales and *o* orientations is given by Eqs (10) and (11).
  $$\begin{array}{c} G_{lo}\left(x,y;f,\theta \right)=\frac{f^{2}}{\pi \gamma \eta }e^{-(\frac{f^{2}}{\gamma^{2}}x^{{}^{\prime }2}+\frac{f^{2}}{\eta^{2}}y^{{}^{\prime }2})}e^{j2\pi fx^{{}^{\prime }}} \end{array}$$ (10)
  $$\begin{array}{c} x^{{}^{\prime }}=xcos\theta +ysin\theta \;\;\;and\;\;\;y^{{}^{\prime }}=-xsin\theta +ycos\theta \end{array}$$ (11)
  Where *f* denotes the central frequency of the filter, *θ* the rotation angle of the Gaussian major axis and the plane wave, *γ* the sharpness along the major axis, and *η* the sharpness along the minor axis.
  The 2D Gabor filter in the frequency domain is given by Eq (12) [30].
  $$\begin{array}{c} \Psi \left(x,y;f,\theta \right)=e^{-\pi^{2}\left(\frac{x^{{}^{\prime }}-f}{\alpha^{2}}+\frac{y^{{}^{\prime }}}{\beta^{2}}\right)} \end{array}$$ (12)
  Where $\alpha =\frac{|f|}{\gamma }$ and $\beta =\frac{|f|}{\eta }$.
  Similarly to wavelets, the multiresolution Gabor descriptor is formed by calculating the energy at every scaled level (see Eq (13)).
  $$\begin{array}{c} Gabor_{l}\left(x,y\right)=\sum_{o=1}^{O}|G_{lo}\left(x,y;f,\theta \right))*I\left(x,y\right)|,\;l\in 1,..,L \end{array}$$ (13)
  The multiresolution Gabor filter, *G*_*lo*_(*f*, *θ*) has been calculated with *L* = 4 scales and *O* = 4 orientations, where I(x,y) represents the image.

### Transformed Space

1. **M-LBP**
  The Local Binary Pattern (LBP) operator is based on the idea that texture is described by patterns or local spatial structures within the image [31]. These patterns may be detected by a 3*x*3 mask called *texture spectrum* that compare masked values, *g*_*p*_(*x*, *y*), with their central pixel, *g*_*c*_(*x*, *y*), acting as threshold. The labeled pixels are multiplied by a fixed weighting function and summed to obtain a value, that is:
  $$\begin{array}{c} LBP_{\left(P,R\right)}\left(x_{c},y_{c}\right)=\sum_{p=0}^{P}H\left(g_{p}\left(x,y\right)-g_{c}\left(x,y\right)\right)2^{p} \end{array}$$ (14)
  Where *g*_*c*_(*p* = 0,.., *P*) are the values of the neighbours and *H*(⋅) is the Heaviside function. (*P*, *R*) are the parameters of the circular mask, with *P* = 8 sampling points and *R* = 1 radius of the neighbourhood. LBP are considered as powerful textural descriptors with discrimination capacity, computational simplicity and tolerance for changes of scale. Several variations have been implemented, such as the improved LBP (ILBP) and the mean LBP (M-LBP). The ILBP uses the mean of all the pixels, $\bar{g_{p}(x,y)}$, instead of using a reference pixel, *g*_*c*_(*x*, *y*) [32]. The M-LBP is similar to the ILBP, but does not consider the central pixel when the binary pattern is created [33]. In this work, the best results were obtained with the M-LBP.
  The formulation of *LBP*_*P*, *R*_ yields a total of 2^*P*^ different patterns. Thus, the range of value pixels is kept between [0…255] to create the texture image and then extract the first and second order Haralick statistical descriptors.
2. **Textons**
  Textons descriptors represent the texture by maps, which are created performing a clusterisation of the principal pixels in an image. There are two types of textons: frequential (F-Textons) and spatial (S-Textons). Frequential textons are based on the responses generated by a filter bank. The filter bank selected is the maximum response filter bank (MR8) composed of a Gaussian and a Laplacian filter, and 18 edge and bar filters with three basic scales. The MR8 filter bank is applied over the tissue images. This process generates 38 response filters per image so that each pixel belongs to the original image and is now represented by a 38 dimensional vector [34]. In the case of spatial textons, they are not filtered by a filter bank, but each image pixel is represented by the intensity values of an NxN square neighbourhood around it. In our study, a 3x3 square neighbourhood was selected. Thus, each original image is now represented by a 9-dimensional vector.
  A k-means clustering algorithm is applied over all the pixel vectors (frequential or spatial). This algorithm allows creating vector groups with similar values. A representative vector called texton is selected for each vector group. Sixty representative textons were selected for each class. Thus, 240 textons were extracted and collected in the texton vocabulary.
  A textons map is generated by each tissue image and the texton vocabulary. These texton maps will be used later as the filtered images used for the classification. The following steps must be done for each image:
  - The MR8 filter bank or the square neighbourhood is applied over the tissue images. Each pixel, now represented by a 38 or a 9-dimensional vector, is assigned to its nearest texton.
  - The texton map is created when all image pixels are classified by their nearest texton. This classification is performed using the k-nearest neighbours algorithm. The texton map is a representation of the original image that assigned the corresponding texton indices (a new colour) to each pixel.
  According to a previous study carried out by the authors of this paper, spatial textons obtained better results in the classification of breast histopahological images stained with HE [12]. For that reason, frequential textons have been dismissed in this study.

### Feature Extraction

**Fig 6:**
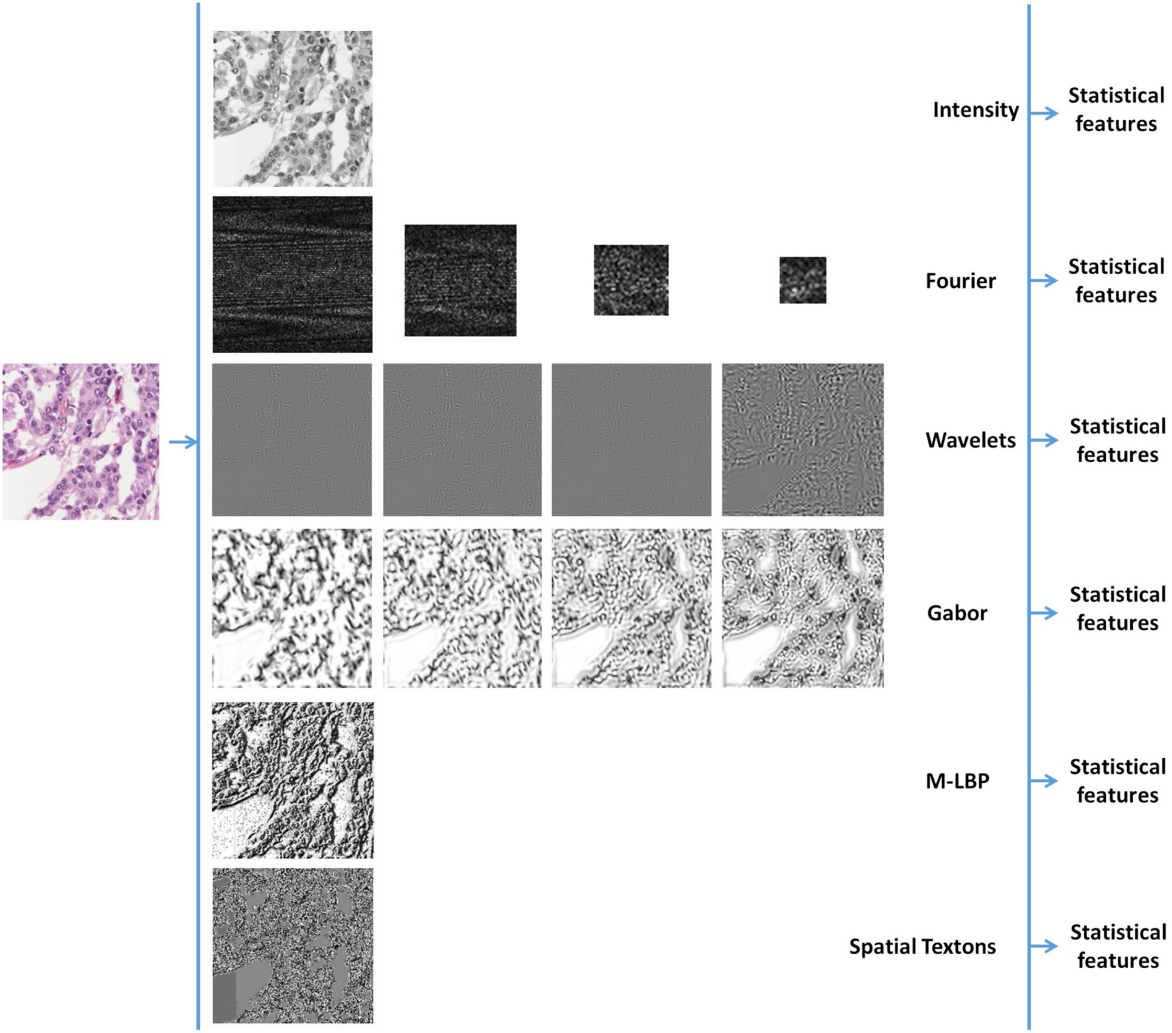
Feature extraction process in a TMA with RGB colour model.

Once the images from our dataset were converted to each colour model they were filtered by each descriptor and the whole bank of statistical descriptors were calculated. Thus, statistical features (Haralick coefficients) were extracted from the original image (called intensity hereafter), each band of Fourier, Wavelets and Gabor, the M-LBP filtered images as well as the spatial texton maps (see Fig 6). This means that the total number of descriptors became four times larger for the space-frequency descriptors, given a four-level decomposition transform. Finally, the first and second order texture statistics are calculated on filtered images of each colour model. Thus, intensity, M-LBP and textons statistical descriptors contain an average of 241 features for each colour model and Fourier, wavelets and Gabor statistical descriptors containing an average of 964 (241*x*4) features. That is, in total for the six textural descriptors, we extract 3615 features for each colour model.

### Dimensionality Reduction

In the correlation method, the dimensionality reduction is estimated by means of a Pearson correlation coefficient, which measures the linear dependence between two or more features. This dependence is estimated by the correlation coefficient [35] shown in the Eq (15), where *cov* is the covariance and *var* the variance.

$$\begin{array}{c} r=\frac{cov(x,y)}{\sqrt{var(x)var(y)}} \end{array}$$ (15)

The Pearson correlation coefficient takes values between 1 and +1. When ∣*r*∣ ≈ 0, this means that there is no correlation (positive or negative) between the features, and x and y are completely uncorrelated. On the other hand, if there is some linear dependence between the x and y features, ∣*r*∣ will take a value near 1.

The correlation method removes redundant and unnecessary information in the feature dataset. In our study, two threshold values of 97% and 99% were adopted for the correlation method. This method achieved an overall 76% dimensionality reduction in our feature dataset maintaining or improving the classification results obtained with the original features.

## Classification

Once textural features are computed, the next issue is how to assign each query case to a pre-established class. Train and classification have been performed using 10-fold cross-validation (10fcv). This validation method divides the feature set into 10 disjoint subsets and performs a loop with 10 iterations using in each iteration nine of the subsets as the training set, and the another subset as the test set. In the end, the average of all folds provides an estimation of the classification accuracy of the model. This type of methodology for classification ensures that each fold has a class distribution similar to the whole dataset. A similar procedure was applied with groups of one element, the so-called *leave-one-out*. Both training methods gave similar results and for the sake of simplicity only 10fcv will be shown in the experiments.

Many classifiers can be applied and some of them could significantly improve accuracy rates. Here, the purpose is to find which descriptor or combination of descriptors better discriminate between breast TMA structures rather than carry out a thorough analysis of classifiers performance. Hence, although we compare an extensive bank of classifiers like nearest-neighbour, k-means, neural networks, decision trees, quadratic Bayes normal classifier, Fisher classifier, linear discriminant or support vector machine (SVM), here, we select five representative ones. That is, the classifiers shown in this manuscript are Fisher, SVM, Random Forest, Bagging and AdaBoost. The classifiers based on weak individual classifiers (tree classifiers in our study) demonstrated a valuable capacity to distinguish the different breast tissue classes.

### Fisher classifier

Fishers linear classifier finds a linear discriminant function by minimising the errors in the least square sense [36]. This linear discriminant is based on finding a direction in the feature space such that the projection of the data minimises Fishers criterion, i.e., the ratio of the squared distance between the class means and averaged class variances. The multi-class implementation used corresponds to the one-against-all strategy.

### Support Vector Machine

Support Vector Machines (SVM) find a discriminant function by maximising the geometrical margin between positive and negative samples [37]. Thus, the space is mapped so that examples from different classes are separated by a gap as wide as possible. Besides linear classification, SVMs can function as a non-linear classifier by using the so-called kernel trick. This trick can be considered a mapping of the inputs onto a high-dimensional feature space in which classes become linearly separable. SVMs minimise both training error and the geometrical margin. The latter accounts for the generalisation abilities of the resulting classifier. In this implementation a linear kernel was used on a multi-class classifier of type one-against-all.

### Random Forest

Random forest was proposed by Breiman [38] as a combination of tree predictors such that each tree depends on the values of a random selection of features. Each decision tree is built using a random subset of the features, independently of the past random subsets but with the same distribution. Finally, the forest chooses the most popular class which is the class with the most votes. The random feature vectors may be generated using several techniques, such as Bagging, random split selection and the so-called random subspace technique.

The forest error rate depends on: i) the strength of the individual trees in the forest and ii) the correlation between two trees in the forest. Random forest algorithm has the advantages of being faster, relatively robust to outliers and noise, giving useful internal estimates of error, strength, correlation and variable importance and easily parallelised. The classifier has been trained using 50 decision trees.

### Bagging Trees

The Bagging (Bootstrap Aggregating) algorithm (see Table 5) is a method of classification that generates weak individual classifiers using Bootstrap. Each classifier is trained on a random redistribution of the training set so many of the original examples may be repeated in each classification [39, 40]. Generally, the error of combining several types of classifiers is explained by bias-variance decomposition. The bias of each classifier is given by its intrinsic error and measures how well a classifier explains the problem. Variance is given by the training set used to create the classifier model. The total error classification is given by the sum of bias and variance. In this paper, the Bagging method was applied to classification trees and ad hoc, an ensemble of 50 trees were taken. The Bagging Tree classifier is the one that obtained the best performance.

**Table 5:** Bagging Algorithm [40].

Training phase
1. Initialize the parameters
-*D* = ∅, the ensemble
-L, the number of classifiers to train
2. For k = 1, …, L
-Take a bootstrap sample *S*_*k*_ from Z.
-Build a classifier *D*_*k*_ using *S*_*k*_ as the training set.
-Add the classifier to the current ensemble, D = D U *D*_*k*_.
3. Return D.
Classification phase
4. Run *D*_*k*_, …, *D*_*L*_ on the input x.
5. The class with the maximum number of votes is chosen as the label for x.

### AdaBoost

AdaBoost was introduced by Freund and Schapire [41] and is based on training different classifiers with different training sets, such as Bagging. The main idea of the algorithm [40] is to assign weights to the training set. Initially, all the weights are equated but each round, the weights of misclassified examples are increased. Thus, in subsequent rounds the weak classifiers will be more focused on these examples (see Table 6). The Adaboost was one of the best classifiers available. As in the random forest classifier, the AdaBoost classifier was trained using 50 decision trees.

**Table 6:** AdaBoost Algorithm [40].

Training phase
1. Initialize the parameters
-Set the weights $w^{1}=[w_{1},...,w_{N}],w_{j}^{1}\in [0,1],\sum_{j=1}^{N}w_{j}^{k}l_{k}^{j}$ (Usually $w_{j}^{1}=\frac{1}{N}$).
-Initialize the ensemble *D* = ∅.
-L, the number of classifiers to train
2. For k = 1, …, L
-Take a sample *S*_*k*_ from Z using distribution *w* ^*k*^.
-Build a classifier *D*_*k*_ using *S*_*k*_ as the training set.
-Calculate the weighted ensemble error at step k by
$\varepsilon_{k}=\sum_{j=1}^{N}w_{j}^{k}l_{k}^{j}$

($l_{k}^{j}=1$ if *D*_*k*_ misclassified *z*_*j*_ and $l_{k}^{j}=0$ otherwise)

-If *ϵ*_*k*_ = 0 or *ϵ*_*k*_ ≥ 0.5, ignore *D*_*k*_, reinitialize the weights $w_{j}^{k}$ to $\frac{1}{N}$ and continue.
-Else, calculate
$\beta_{k}=\frac{\epsilon_{k}}{1-\epsilon_{k}}$, where *ϵ*_*k*_ ∈ (0,0.5)

-Update the individual weights
$w_{j}^{k+1}=\frac{w_{j}^{k}\beta_{k}^{\left(1-l_{k}^{j}\right)}}{\sum_{i=1}^{N}w_{i}^{k}\beta_{k}^{(1-l_{k}^{j}}}$, j = 1, …, N.

3. Return D and *β*_1_, …, *β*_*L*_.
Classification phase
4. Calculate the support for class *ω*_*t*_ by
$\mu_{t}(x)=\sum_{D_{k}=\omega_{t}}ln(\frac{1}{\beta_{k}})$
5. The class with the maximum support is chosen as the label for x.

## Evaluation Metrics

An important aspect when diagnostic results are managed is their correct interpretation. In classification problems there are two types of results produced: positive and negative detections or classifications. However, in some cases, positive types can be classified as negative and vice versa. These cases are called false positives and false negatives, respectively. Thus, four types of outputs, that is, true positive (TP), true negative (TN), false positive (FP) and false negative (FN) must be considered in the interpretation of results.

The following validity and security criteria are fulfilled when the classification results are interpreted:

- Validity denotes the grade of validity over the results. This condition is measured by the sensitivity and specificity coefficients (see Eq (16)). The *sensitivity* represents the conditional probability of classifying the tissue as positive. Then, this coefficient indicates the capability that the selected classifier has to detect positive cases. The *specificity* shows the conditional probability of classifying the tissue as negative. Therefore, in contrast to the sensitivity, the specificity indicates the capability that the classifier has of detecting the negative cases.
  $$\begin{array}{c} Sensitivity=\frac{TP}{TP+FN}\;\;\;\;\;\;\;\;Specificity=\frac{TN}{TN+FP} \end{array}$$ (16)
- Security is determined by the predictive value of the result (see Eq (17)). What is the security level in the classification result to predict negative or positive cases? The *positive predictive value* (PPV) or *precision* indicates the likelihood of a positive case being classified as such. This value is estimated using the amount of real positive cases that were classified as positive. The *negative predictive value* (NPV) indicates the likelihood that a negative case had been classified as such. In the same ways as the previous predictive value, it is estimated using the amount of real negative cases that were classified as negative. Finally, the *accuracy* (ACC) is the proportion of true results (true positives and true negatives).
  $$\begin{array}{c} PPV=\frac{TP}{TP+FP}\;\;\;\;\;\;NPV=\frac{TN}{TN+FN}\;\;\;\;\;\;ACC=\frac{TP+TN}{TP+FP+FN+TN} \end{array}$$ (17)

The evaluation metrics used in this paper are based on the binary classification method. In multi-class problems, the metrics are calculated taking the values of a single class against the mean values in all other classes (one-vs.-rest) [42].

### ROC Curves

The ROC (Receiver operating characteristic) curve is another common technique to validate results [43]. Basically, it consists of a graphic plot that represents the sensitivity versus (1-specificity) for a binary classifier system, and therefore, the diagnostic accuracy of the test. ROC curves have several purposes:

- To determine the point where greater sensitivity and specificity is achieved.
- To evaluate the discriminative ability of the test
- To compare this capability among several different tests

The Youden index is the point that represents the largest sensitivity and specificity. Graphically, it is the nearest point to the coordinate (0,1) (upper-left corner of the graph). The discriminative ability of the test is its capability to distinguish between positive and negative cases. This capability is measured by the area under the curve (AUC). This area has a value between 0.5 and 1, where 1 represents a perfect diagnosis and 0.5 a test without a discriminating capacity.

## Empirical Results

Classification results depend on feature quality and the suitable selection of the classifier. Several experiments were done to show concrete aspects of descriptors and classifiers. As mentioned above, in this study, a relevant set of features was obtained from first and second order Haralick statistical descriptors obtained from the intensity image, Fourier, Wavelets, Gabor, M-LBP and spatial texton descriptors. Furthermore, each feature set was extracted for each colour space discussed in previous sections that is, RGB, CMYK, HSV, Lab, Luv, SCT and channel combinations Lb and Hb. However, the combination of so many colour models and descriptors can substantially increase the size of the feature set; hence, the importance of using methods to reduce feature dimensionality.

The combination of different features and classifiers implies a considerable number of possible experiments. In order to perform all of these tests, four types of classification experiments were performed with six classifiers: (1) classification per colour model individually, (2) classification by combination of colour models, (3) classification by combination of colour models and descriptors, and (4) classification by combination of colour models and descriptors with a previous feature set reduction.

### Experiment 1: Results per colour model

**Fig 7:**
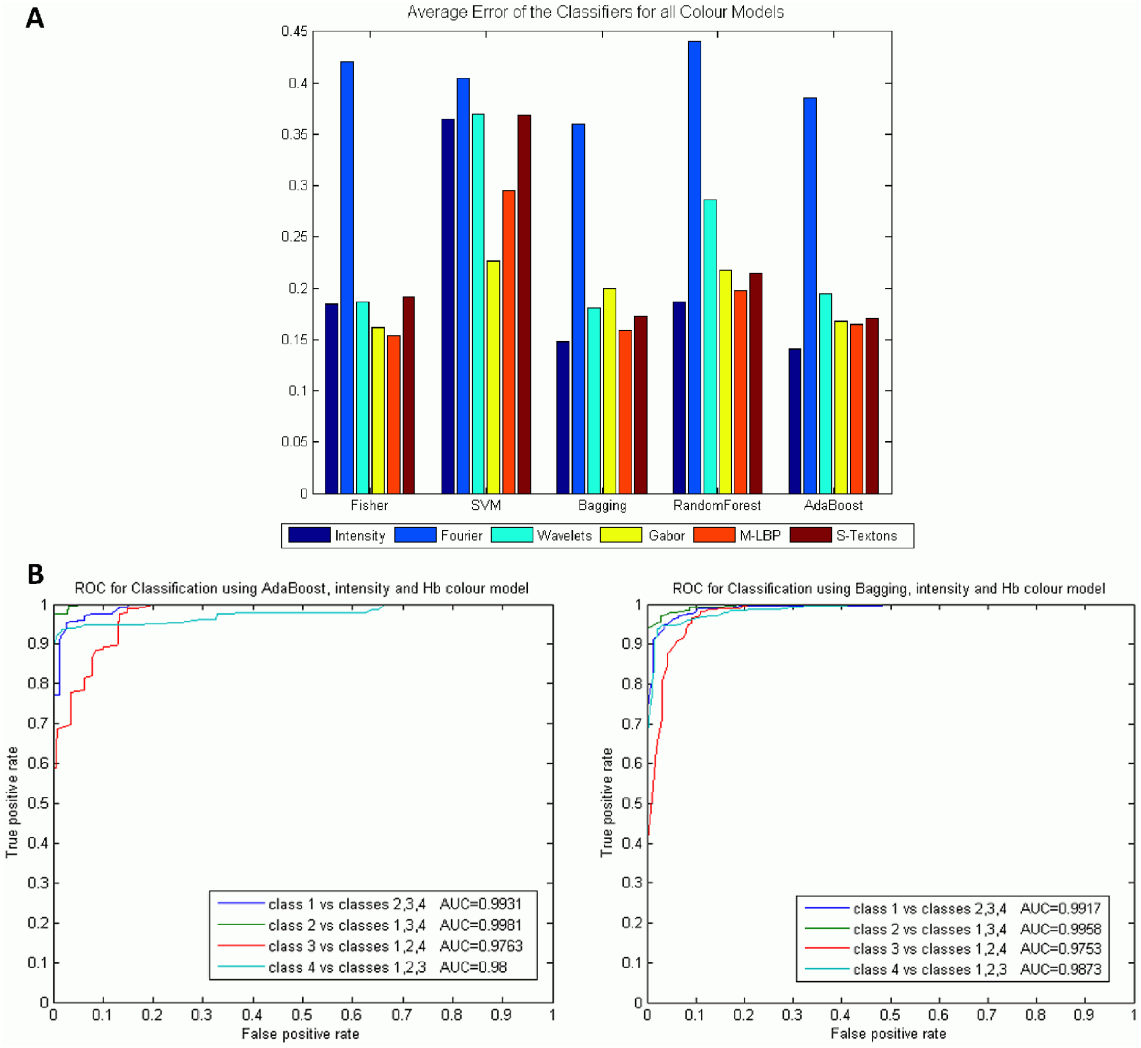
Results obtained using colour models independently. A) Average error of all classifiers and descriptors, B) ROC curves for Classification using AdaBoost and Bagging with intensity and Hb colour model.

As mentioned above, some colour models are able to highlight different breast tissue structures, and therefore, improve the classification results. That will be the first improvement in our study. A deep study of the different colour models and their combination was done. On average the best classifiers that work well with all features and colour models are the AdaBoost and the Bagging Trees with an average error of 0.18. The error refers to mislabelling in the 4-class classification. Reviewing the results individually, the best performance was achieved with the intensity statistical features applied to the Hb image and using AdaBoost, with an error of 0.061, an average of 93.92% PPV and 97% ACC. The worst classification error was obtained by the statistical Fourier descriptor. Fig 7a shows the average results of all the classifiers and descriptors applied to each colour model, and Fig 7b shows the ROC curves of the first and second best classifier results, AdaBoost and Bagging Trees, applied to intensity and Hb colour model. These are the classifiers with the best global results for colour model and descriptor.

### Experiment 2: Results of combining colour models

An important question is whether the combination of colour models can improve subsequent classification results. To this end, all the features of each colour model were combined in the same feature dataset and again, a classification with the same classifiers was carried out. Thus, intensity, M-LBP and S-Textons statistical descriptors multiplied their features by 8 (colour models analysed) with a total average of 1928 features. On the other hand, up to 7712 features were used with Fourier, Wavelets and Gabor (for each descriptor).

**Fig 8:**
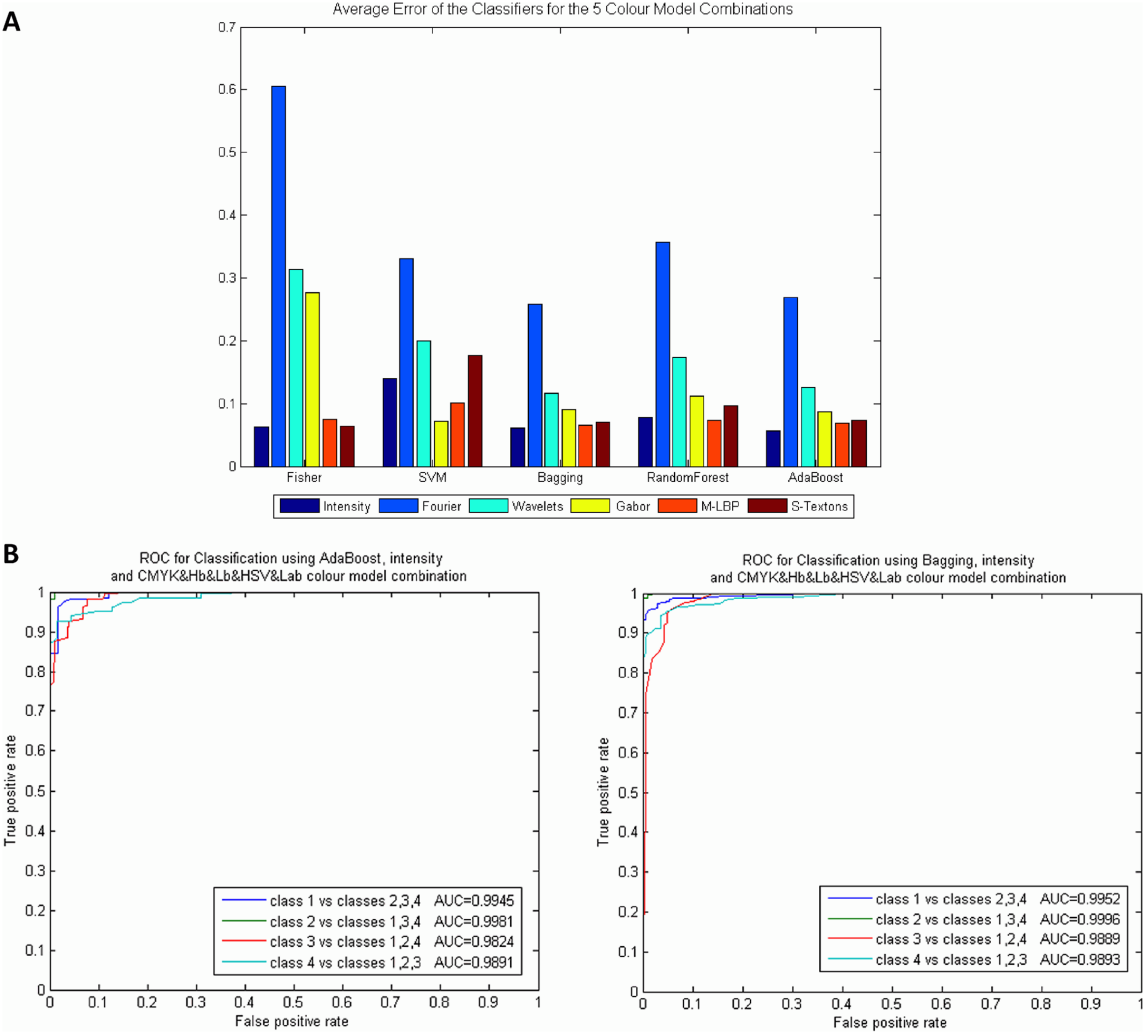
Results obtained using colour model combinations. A) Average error of all classifiers and descriptors, B) ROC curves for Classification using AdaBoost and Bagging with intensity and CMYK&Hb&Lb&HSV&Lab colour model combination.

It was observed that the results improved significantly when more than three colour models were combined. In fact, five colour model combinations provide better results; they were: Hb&Luv&SCT, CMYK&Hb&Lb&HSV&Lab, CMYK&Hb&Lb&HSV&Luv&SCT, RGB&Hb&Lb&HSV&Luv&SCT and the eight colour models. On average the best classifier that works well with all features and all colour model combinations is the AdaBoost Bagging Tree with an average error of 0.11. Reviewing the results individually, the best performance was achieved with the Fisher and AdaBoost classifiers by means of the M-LBP and intensity statistical descriptors with the Hb&Luv&SCT and CMYK&Hb&Lb&HSV&Lab colour model combinations, respectively. Both Fisher and AdaBoost classifiers obtained an error of 0.049 with an average of 95.07 and 95.18% PPV, and 97.39% and 97.81% ACC, respectively. The computational time was 20.26 seconds for Fisher and 123.45 seconds for Adaboost classification. The worst classification error was also obtained by the statistical Fourier and Wavelet descriptors and with the SVM. Fig 8a shows the average results of all the classifiers and descriptors applied to the combination of the colour model, and Fig 8b shows the ROC curves of the best results obtained using the AdaBoost and Bagging classifiers respectively applied to intensity with the CMYK&Hb&Lb&HSV&Lab colour model combination.

### Experiment 3: Results of combining colour models and descriptors

The objective of this experiment is to confirm whether the colour model and descriptor combination improves the classification results even more. For that reason, the best descriptors of Experiment 2 were combined. Six combinations of descriptors were tested: Intensity&M-LBP which make 482 features, Intensity&S-Textons (482 features), Intensity&M-LBP&Gabor (1446 features), Intensity&M-LBP&S-Textons (723 features), Intensity&M-LBP&Gabor&S-Textons (1687 features) and Intensity&M-LBP&Gabor&Wavelets (2410 features). Thus, the number of descriptors ranges from 482 to 2410 per three, five, six and eight colour models used in the five colour model combinations.

**Fig 9:**
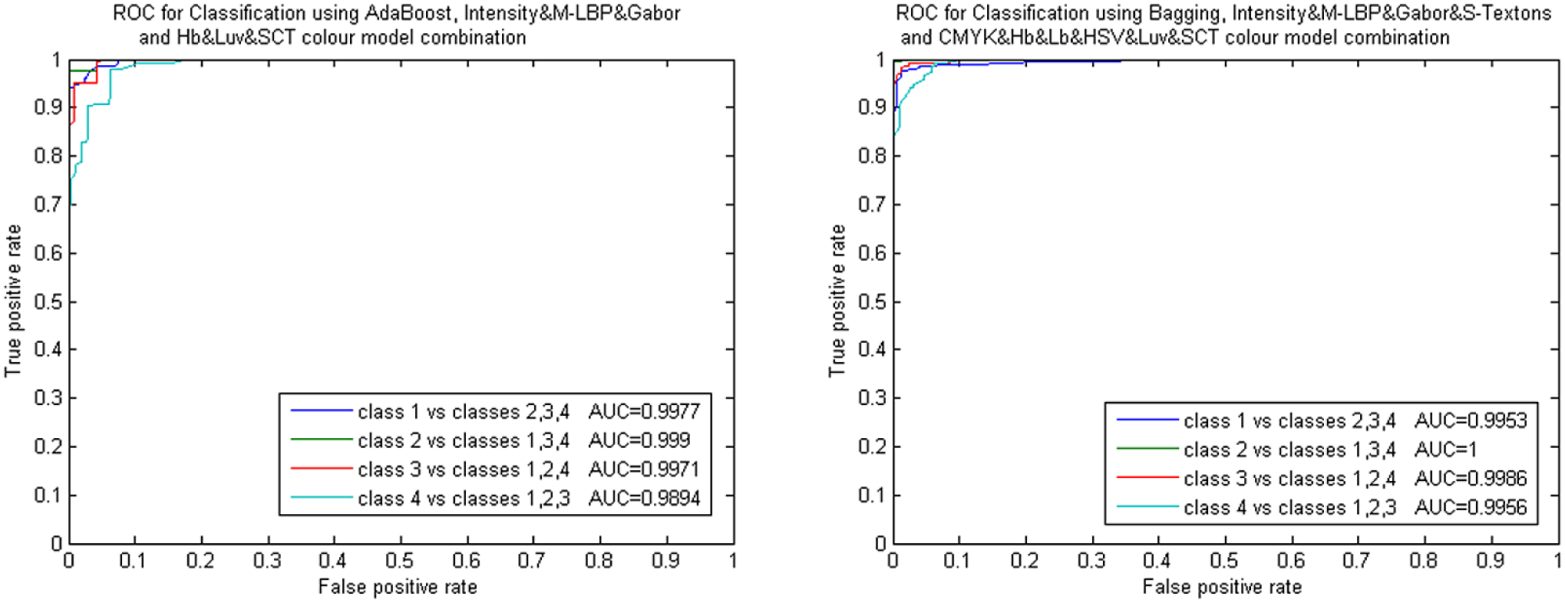
ROC curves for Classification using AdaBoost and Bagging. AdaBoost results with Intensity&M-LBP&Gabor and Hb&Luv&SCT colour model combination. Bagging results with Intensity&M-LBP&Gabor&S-Textons and CMYK&Hb&Lb&HSV&Luv&SCT colour model combination.

Once again, the AdaBoost and the Bagging Tree classifiers were the best classifiers. Bagging classifier using CMYK&Hb&Lb&HSV&Luv&SCT colour combination with the Intensity&M-LBP&Gabor&S-Textons descriptors, and AdaBoost classifier using the Hb&Luv&SCT colour combination with Intensity&M-LBP&Gabor descriptors. The ROC curves are shown in Fig 9. The best result was obtained with the Bagging classifier, and the confusion matrix is shown in Table 7. The results are promising, reaching 98.26% ACC and 96.95% PPV, and taking 181.92 seconds for the classification. It is clear that the Bagging classifier is the most powerful classifier with our feature dataset.

**Table 7:** Best classification using a Bagging classifier and a combination of CMYK&Hb&Lb&HSV&Lab colour models and Intensity&M-LBP&Gabor&S-Textons descriptors.

Label	E1	E2	E3	E4	PPV	NPV	Sensitivity	Specificity	ACC
1	168	0	1	1	98.8	97.4	93.3	99	97.77
2	0	102	0	1	99	99.8	99	99.8	99.68
3	5	0	157	1	96.3	98.9	96.9	98.7	98
4	7	1	4	180	93.7	99.3	98	97.3	97.6

### Experiment 4: Results of combining colour models and descriptors with a prior reduction of correlated features

Although the previous experiments improve the classification results, the combination of colour models and descriptors also increases the number of features, and thus, the computation time needed. In addition, some features may be redundant or provide irrelevant information getting worse results. Irrelevant and redundant features could be detrimental for the training processes and increase the computational time [44]. New feature sets smaller than the originals and without redundant information may be created. As mentioned above, the correlation method [35] was analysed to carry out the dimensionality reduction.

Correlation was performed with two threshold values, 97% and 99%, which allow a reduction of 75.73% and 75.79% of the initial features, respectively.

**Fig 10:**
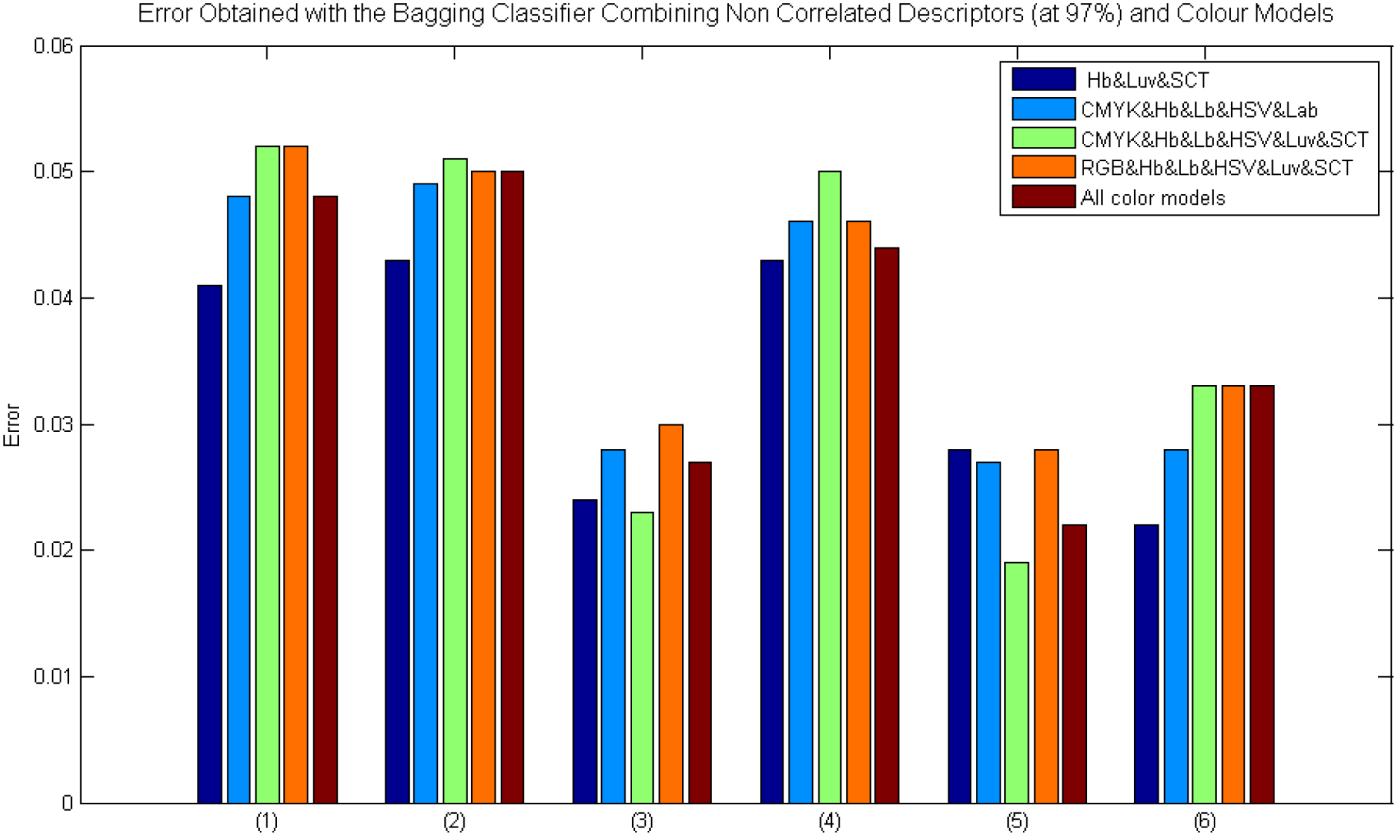
Results using the Bagging classifier with colour model and feature combinations and a previous correlation analysis using a threshold of 97%. Where: (1) Intensity&M-LBP, (2) Intensity&S-Textons, (3) Intensity&M-LBP&Gabor, (4) Intensity&M-LBP&S-Textons, (5) Intensity&M-LBP&Gabor&S-Textons and (6) Intensity&M-LBP&Gabor&Wavelets.

It has to be pointed out that the best results were obtained by Bagging Tree classifier and a correlation threshold of 97% (see Fig 10). Note that using Intensity&M-LBP&Gabor, Intensity&M-LBP&Gabor&S-Textons, and Intensity&M-LBP&Gabor& Wavelets descriptor combinations the error value is lower than 0.035.

**Fig 11:**
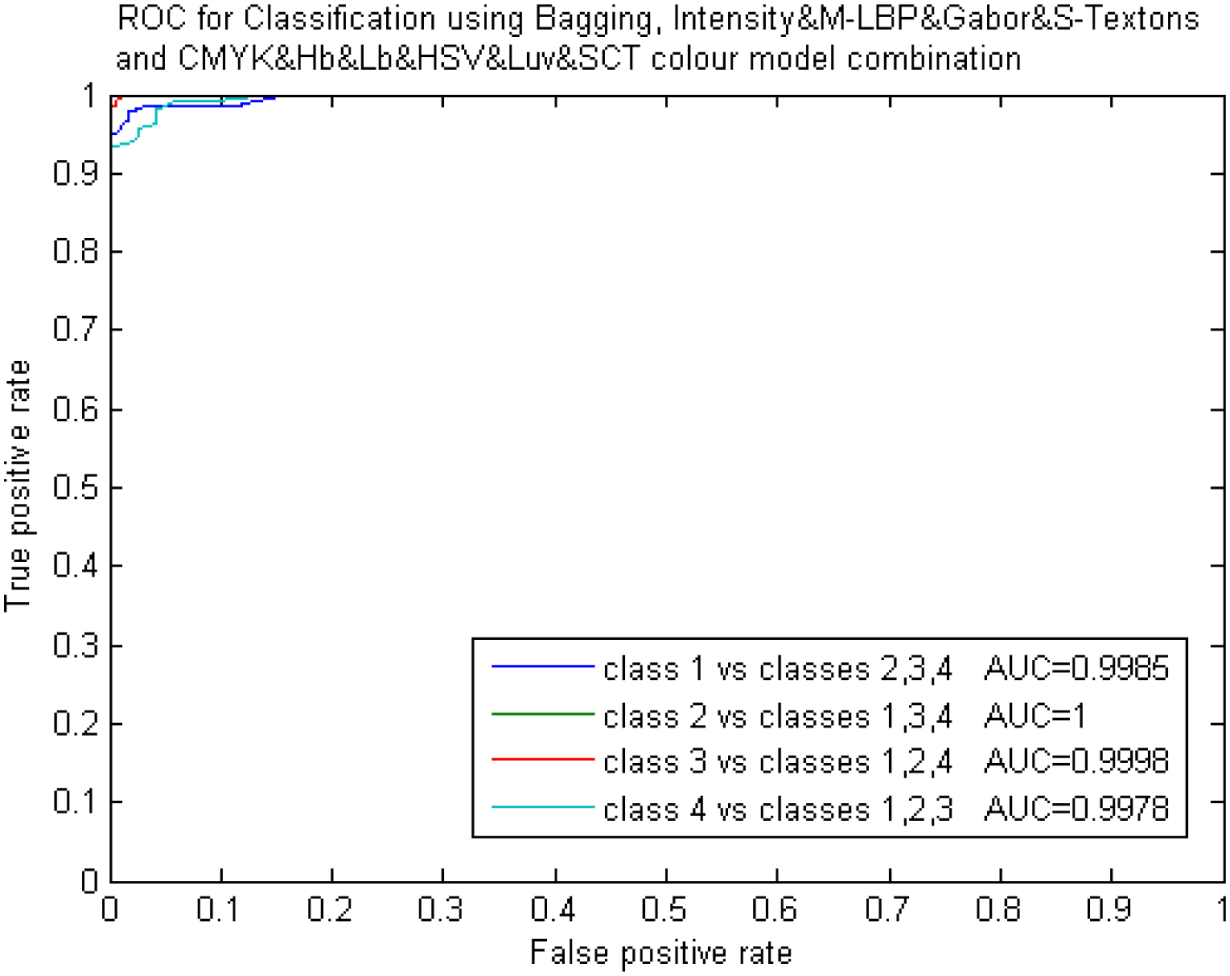
ROC curves for the best result in Experiment 4 (Non correlated features at 97%).

In this experiment, the best result was obtained with the CMYK&Hb&Lb&HSV&Luv&SCT colour combination and Intensity&M-LBP&Gabor&S-Textons descriptors. Confusion matrices are shown in Table 8 where an average of 99.05% ACC and 98.34% PPV were obtained with a total of 1719 features. ROC curves are shown in Fig 11. The computational time of the classification was 244.2 seconds. Thus, the validity of the colour model and descriptor combination in the breast TMA classification is demonstrated.

**Table 8:** The best final classification was obtained by a previous correlation threshold of 97% and the Bagging classifier combining CMYK&Hb&Lb&HSV&Luv&SCT colour model and Intensity&M-LBP&Gabor&S-Textons descriptors.

Label	Total	E1	E2	E3	E4	PPV	NPV	Sensitivity	Specificity	ACC
1	170	166	0	1	3	97.65	98.47	95.9	99.12	98.25
2	103	0	103	0	0	100	100	100	100	100
3	163	1	0	162	0	99.38	99.57	98.78	99.78	99.52
4	192	6	0	1	185	96.35	99.31	98.4	98.41	98.41

## Discussion

This paper has described a complete study on breast TMA classification using the combination of colour and texture descriptors. The study shows promising results with a dataset of 628 TMA images divided into four classes, including benign anomalous structures, which are ignored in most studies. Overall, class 2, that is, adipose tissue, is the class best classified while classes 1 (benign stromal tissue with cellularity) and 4 (ductal and lobular carcinomas) show greater difficulty in the classification. Nevertheless, a suitable combination of descriptors and colour models makes our results obtain an error value below 0.035. Besides, different dimensionality reduction methods were performed due to the large size of the feature dataset and the increase of the computational time. Finally, the best result was obtained with the combination of intensity, M-LBP, Gabor and S-Textons statistical descriptors and a suitable combination of colour models with CMYK, Hb, Lb, HSV, Luv and SCT reducing correlated features with a 97% correlation threshold and using a Bagging tree classifier. This test obtained an average of 99.05% accuracy and 98.34% positive predictive value making this study truly valuable in breast TMA classification. In addition, although the number of features was large, computational times in the classification were not very excessive, and therefore, the CAD methodology proposed is suitable for the daily work of pathologists.

## Appendix I

This section has been added in order to evaluate the results of the two best classifiers, the AdaBoost and Bagging Tree classifiers. Therefore, the purpose of this appendix is to carry out a comparison between the classifiers which have given the best overall results by experiment. These results will be ordered by the type of colour and descriptor combination. Let us remind the four experiments performed: (1) Classification per colour model individually, (2) Classification by combination of colour models and (3) Classification by combination of colour models and descriptors and (4) Classification by combination of colour models and descriptors with a previous feature set reduction.

### AdaBoost and Bagging classifiers in Experiment 1 results

**Fig 12:**
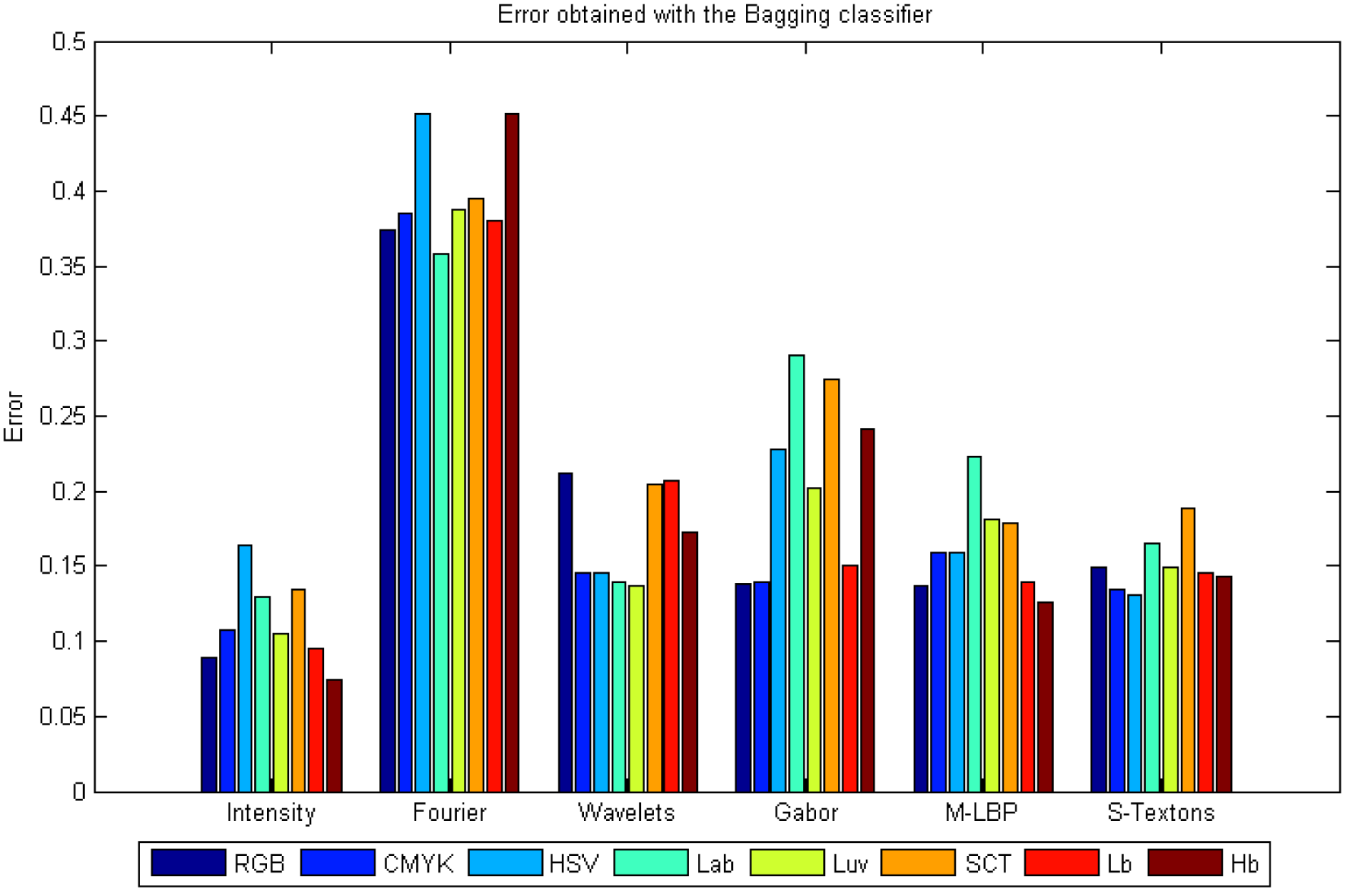
Results obtained from the Bagging classifier using colour models independently.

**Fig 13:**
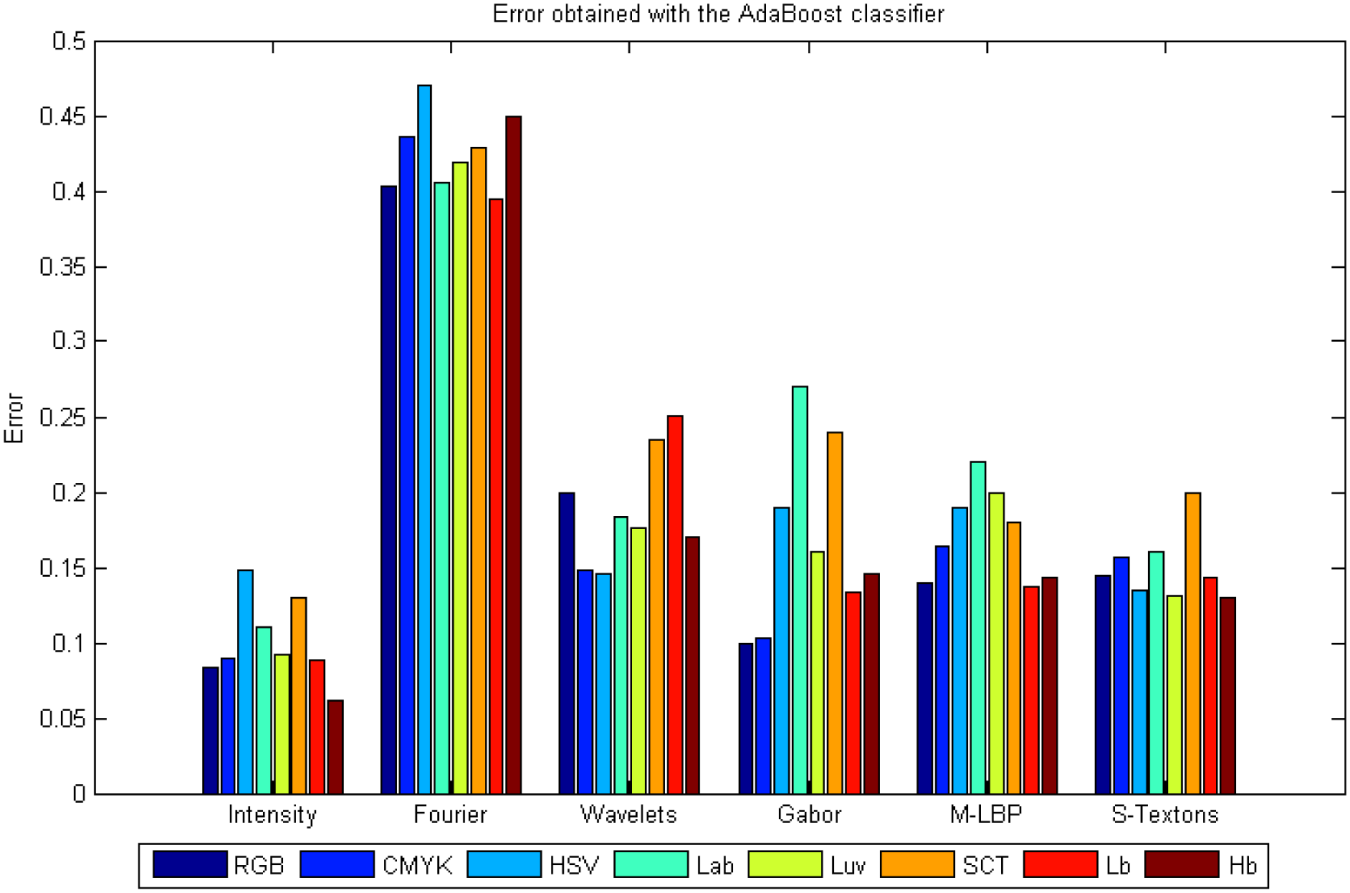
Results obtained from the AdaBoost classifier using colour models independently.

Results obtained in this experiment are shown in Figs 12 and 13.

### AdaBoost and Bagging classifiers in Experiment 2 results

**Fig 14:**
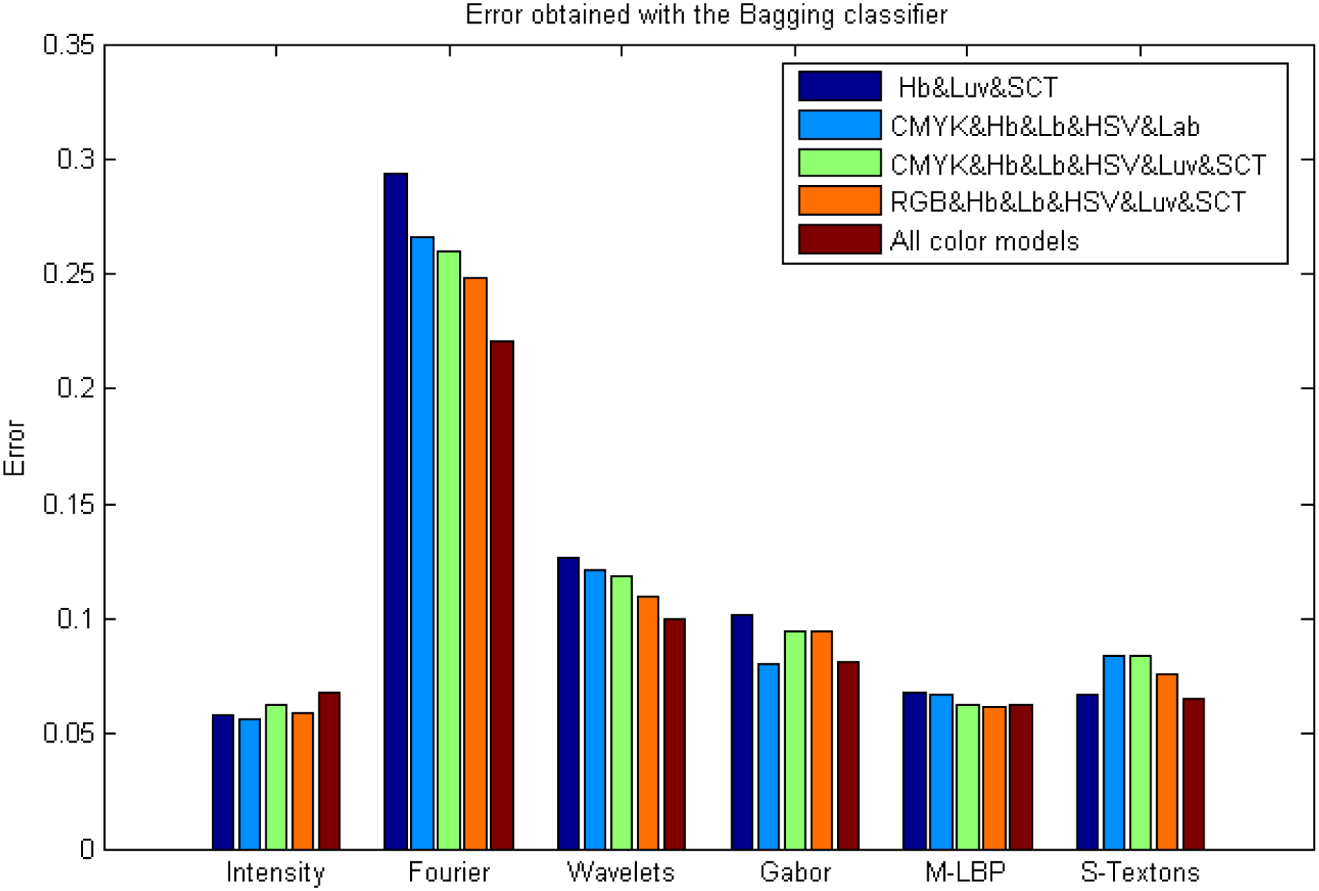
Results obtained from the Bagging classifier using colour model combination.

**Fig 15:**
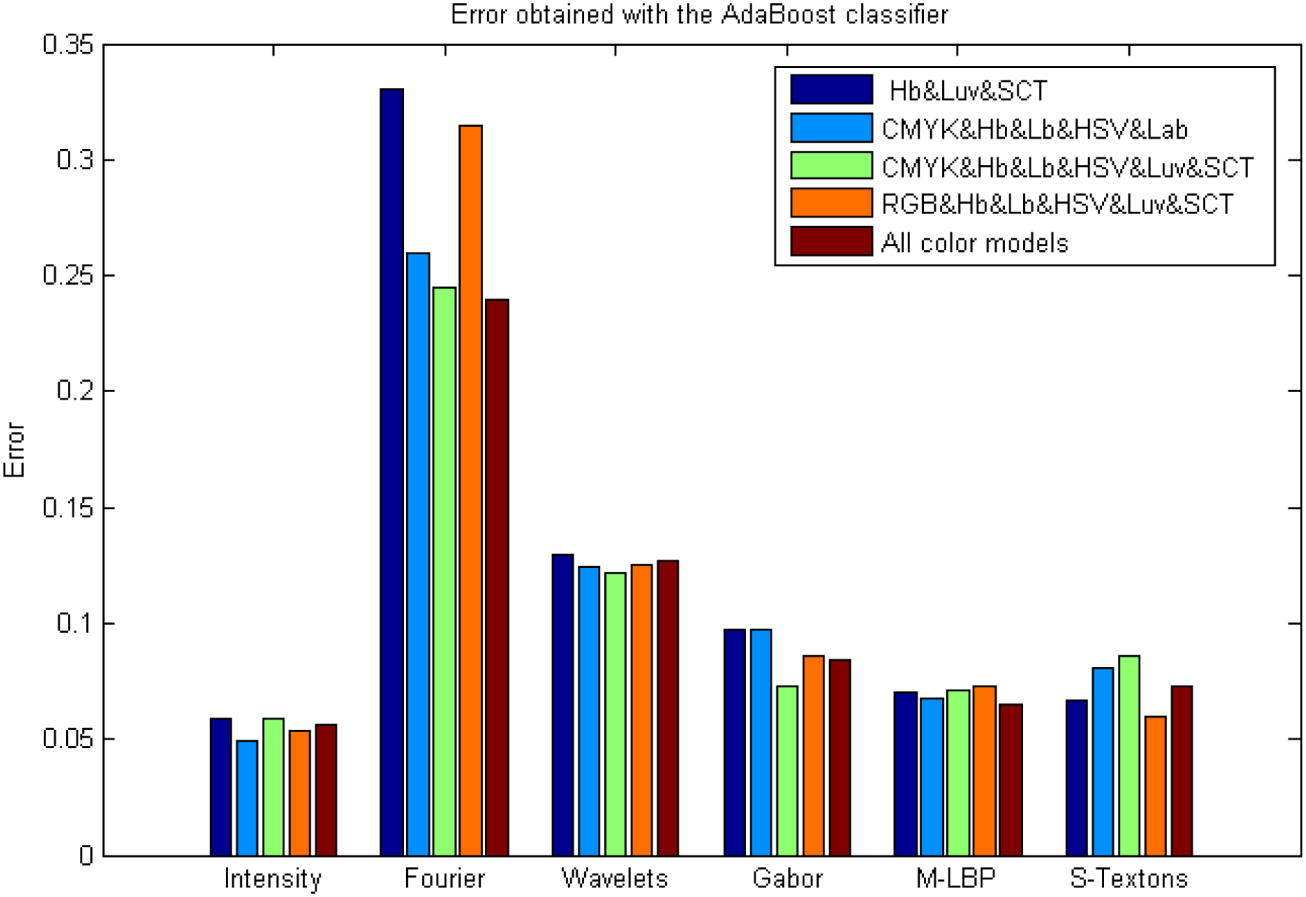
Results obtained from the AdaBoost classifier using colour model combination.

Although in this experiment the best classification results were obtained with Fisher and AdaBoost classifiers, the third best result by classifier is obtained by the Bagging classifier achieving an error of 0.056 using intensity statistical descriptors with the Hb&Luv&SCT colour model combinations. Classification errors are shown in Figs 14 and 15, respectively.

### AdaBoost and Bagging classifiers in Experiment 3 results

**Fig 16:**
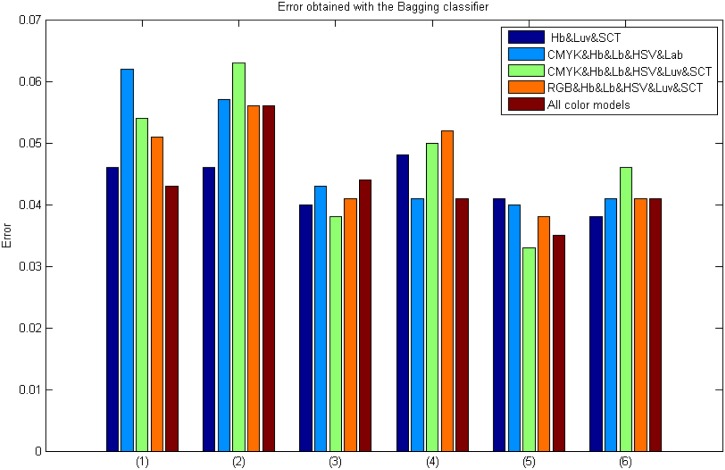
Results using the Bagging classifier with a combination of colour models and descriptors. Where: (1) Intensity&M-LBP, (2) Intensity&S-Textons, (3) Intensity&M-LBP&Gabor, (4) Intensity&M-LBP&S-Textons, (5) Intensity&M-LBP&Gabor&S-Textons and (6) Intensity&M-LBP&Gabor&Wavelets.

**Fig 17:**
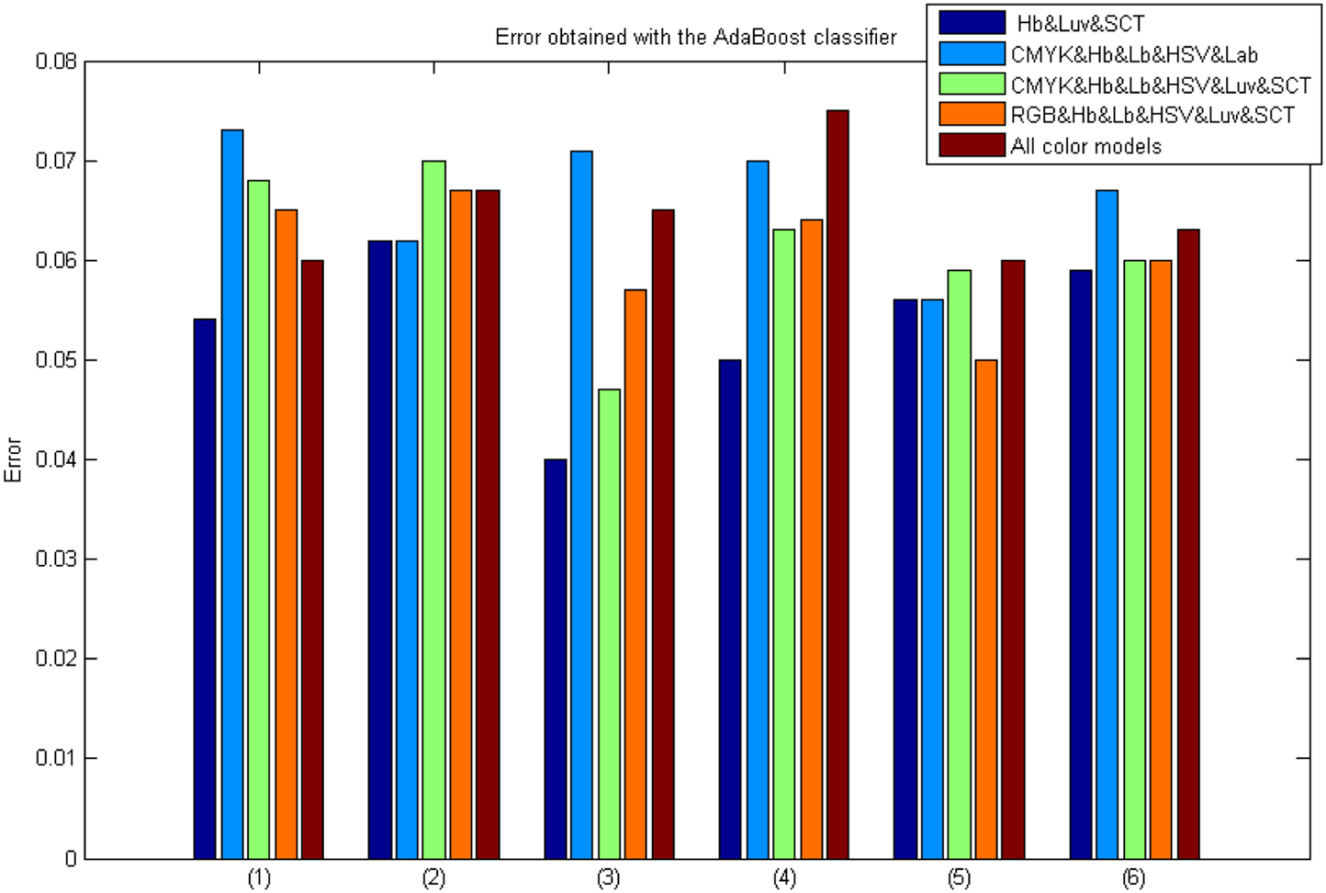
Results using the AdaBoost classifier with a combination of colour models and descriptors. Where: (1) Intensity&M-LBP, (2) Intensity&S-Textons, (3) Intensity&M-LBP&Gabor, (4) Intensity&M-LBP&S-Textons, (5) Intensity&M-LBP&Gabor&S-Textons and (6) Intensity&M-LBP&Gabor&Wavelets.

Figs 16 and 17 show the classification errors obtained during this experimentation.

### AdaBoost and Bagging classifiers in Experiment 4 results

**Fig 18:**
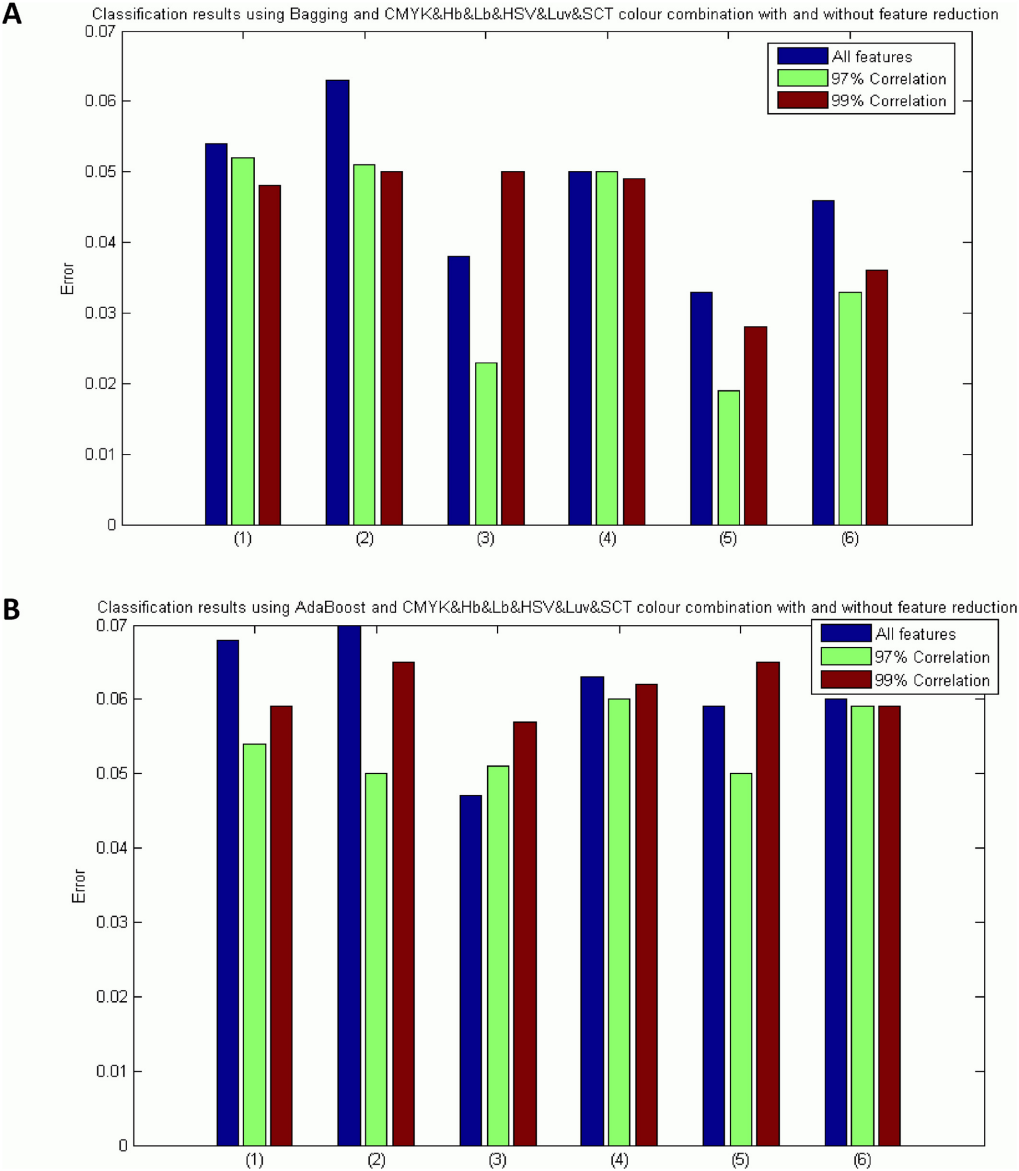
Results using the best classifier with the best combination of colour models and descriptors with and without feature selection. Where: (1) Intensity&M-LBP, (2) Intensity&S-Textons, (3) Intensity&M-LBP&Gabor, (4) Intensity&M-LBP&S-Textons, (5) Intensity&M-LBP&Gabor&S-Textons and (6) Intensity&M-LBP&Gabor&Wavelets. A) Bagging Tree Classifier, B) AdaBoost Classifier.

Tables 9 and 10 show the results obtained depending on the correlation threshold value selected. In addition, a comparison between the results with and without feature selection is shown in Fig 18a and 18b.

**Table 9:** Classification using a combination of colour models and descriptors and a 97% correlation threshold.

		Bagging	AdaBoost
Hb&Luv&SCT	Intensity&M-LBP	0.041	0.051
	Intensity&S-Textons	0.043	0.059
	Intensity&M-LBP&Gabor	0.024	0.033
	Intensity&M-LBP&S-Textons	0.043	0.056
	Intensity&M-LBP&Gabor&S-Textons	0.028	0.041
	Intensity&M-LBP&Gabor&Wavelets	0.022	0.048
CMYK&Hb&Lb& HSV&Lab	Intensity&M-LBP	0.048	0.057
	Intensity&S-Textons	0.049	0.06
	Intensity&M-LBP&Gabor	0.028	0.051
	Intensity&M-LBP&S-Textons	0.046	0.057
	Intensity&M-LBP&Gabor&S-Textons	0.027	0.054
	Intensity&M-LBP&Gabor&Wavelets	0.028	0.047
CMYK&Hb&Lb&HSV&Luv&SCT	Intensity&M-LBP	0.052	0.054
	Intensity&S-Textons	0.051	0.05
	Intensity&M-LBP&Gabor	0.023	0.051
	Intensity&M-LBP&S-Textons	0.05	0.06
	Intensity&M-LBP&Gabor&S-Textons	0.019	0.05
	Intensity&M-LBP&Gabor&Wavelets	0.033	0.059
RGB&Hb&Lb&HSV&Luv&SCT	Intensity&M-LBP	0.052	0.062
	Intensity&S-Textons	0.05	0.063
	Intensity&M-LBP&Gabor	0.03	0.056
	Intensity&M-LBP&S-Textons	0.046	0.063
	Intensity&M-LBP&Gabor&S-Textons	0.028	0.054
	Intensity&M-LBP&Gabor&Wavelets	0.033	0.054
All colour models	Intensity&M-LBP	0.048	0.059
	Intensity&S-Textons	0.05	0.054
	Intensity&M-LBP&Gabor	0.027	0.052
	Intensity&M-LBP&S-Textons	0.044	0.062
	Intensity&M-LBP&Gabor&S-Textons	0.022	0.05
	Intensity&M-LBP&Gabor&Wavelets	0.033	0.052

**Table 10:** Classification using a combination of colour models and descriptors and a 99% correlation threshold.

		Bagging	AdaBoost
Hb&Luv&SCT	Intensity&M-LBP	0.044	0.048
	Intensity&S-Textons	0.043	0.05
	Intensity&M-LBP&Gabor	0.035	0.043
	Intensity&M-LBP&S-Textons	0.04	0.052
	Intensity&M-LBP&Gabor&S-Textons	0.027	0.06
	Intensity&M-LBP&Gabor&Wavelets	0.025	0.044
CMYK&Hb&Lb& HSV&Lab	Intensity&M-LBP	0.052	0.067
	Intensity&S-Textons	0.057	0.054
	Intensity&M-LBP&Gabor	0.028	0.05
	Intensity&M-LBP&S-Textons	0.059	0.063
	Intensity&M-LBP&Gabor&S-Textons	0.033	0.056
	Intensity&M-LBP&Gabor&Wavelets	0.028	0.048
CMYK&Hb&Lb&HSV&Luv&SCT	Intensity&M-LBP	0.048	0.059
	Intensity&S-Textons	0.05	0.065
	Intensity&M-LBP&Gabor	0.033	0.057
	Intensity&M-LBP&S-Textons	0.049	0.062
	Intensity&M-LBP&Gabor&S-Textons	0.028	0.065
	Intensity&M-LBP&Gabor&Wavelets	0.036	0.059
RGB&Hb&Lb&HSV&Luv&SCT	Intensity&M-LBP	0.051	0.067
	Intensity&S-Textons	0.056	0.062
	Intensity&M-LBP&Gabor	0.041	0.05
	Intensity&M-LBP&S-Textons	0.044	0.062
	Intensity&M-LBP&Gabor&S-Textons	0.028	0.054
	Intensity&M-LBP&Gabor&Wavelets	0.043	0.057
All colour models	Intensity&M-LBP	0.049	0.063
	Intensity&S-Textons	0.054	0.056
	Intensity&M-LBP&Gabor	0.04	0.052
	Intensity&M-LBP&S-Textons	0.043	0.067
	Intensity&M-LBP&Gabor&S-Textons	0.036	0.057
	Intensity&M-LBP&Gabor&Wavelets	0.042	0.056

## Supporting Information

S1 Database Files RGB tissue images used in this paper. (ZIP)
