# Supplementary figures and images for: Influence of Texture and Colour in Breast TMA Classification (part 2 of 2)
Source: PLoS One. 2015 Oct 29;10(10):e0141556. doi: 10.1371/journal.pone.0141556 (PMC4626403; doi:10.1371/journal.pone.0141556)

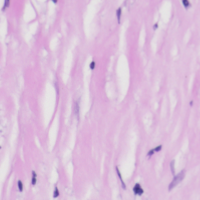

Supplement: S1 Database Files — (ZIP) [file pone.0141556.s001.zip › DATABASE/CLASS1TIF/zona28.tif]

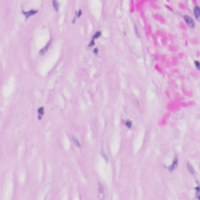

Supplement: S1 Database Files — (ZIP) [file pone.0141556.s001.zip › DATABASE/CLASS1TIF/zona29.tif]

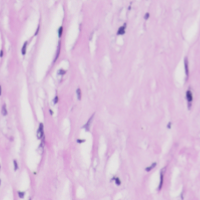

Supplement: S1 Database Files — (ZIP) [file pone.0141556.s001.zip › DATABASE/CLASS1TIF/zona30.tif]

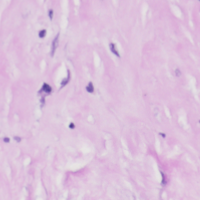

Supplement: S1 Database Files — (ZIP) [file pone.0141556.s001.zip › DATABASE/CLASS1TIF/zona31.tif]

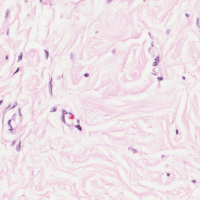

Supplement: S1 Database Files — (ZIP) [file pone.0141556.s001.zip › DATABASE/CLASS1TIF/zona32.tif]

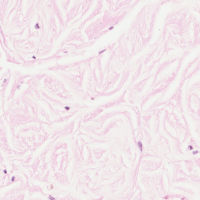

Supplement: S1 Database Files — (ZIP) [file pone.0141556.s001.zip › DATABASE/CLASS1TIF/zona33.tif]

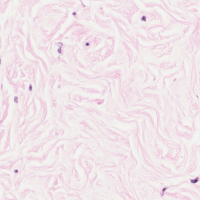

Supplement: S1 Database Files — (ZIP) [file pone.0141556.s001.zip › DATABASE/CLASS1TIF/zona34.tif]

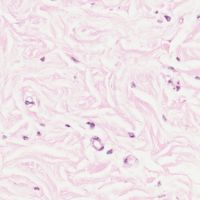

Supplement: S1 Database Files — (ZIP) [file pone.0141556.s001.zip › DATABASE/CLASS1TIF/zona35.tif]

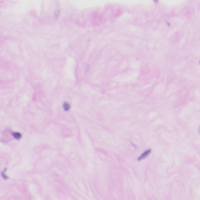

Supplement: S1 Database Files — (ZIP) [file pone.0141556.s001.zip › DATABASE/CLASS1TIF/zona36.tif]

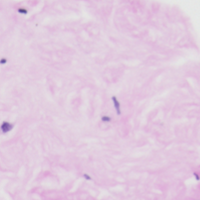

Supplement: S1 Database Files — (ZIP) [file pone.0141556.s001.zip › DATABASE/CLASS1TIF/zona37.tif]

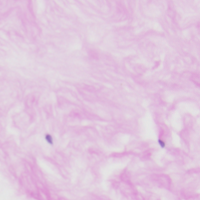

Supplement: S1 Database Files — (ZIP) [file pone.0141556.s001.zip › DATABASE/CLASS1TIF/zona38.tif]

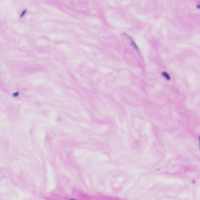

Supplement: S1 Database Files — (ZIP) [file pone.0141556.s001.zip › DATABASE/CLASS1TIF/zona39.tif]

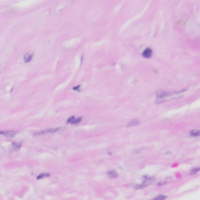

Supplement: S1 Database Files — (ZIP) [file pone.0141556.s001.zip › DATABASE/CLASS1TIF/zona40.tif]

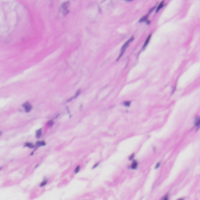

Supplement: S1 Database Files — (ZIP) [file pone.0141556.s001.zip › DATABASE/CLASS1TIF/zona41.tif]

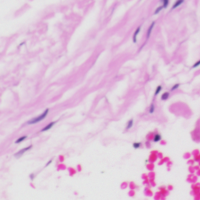

Supplement: S1 Database Files — (ZIP) [file pone.0141556.s001.zip › DATABASE/CLASS1TIF/zona42.tif]

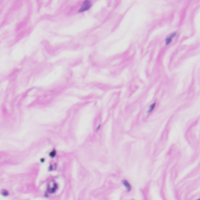

Supplement: S1 Database Files — (ZIP) [file pone.0141556.s001.zip › DATABASE/CLASS1TIF/zona43.tif]

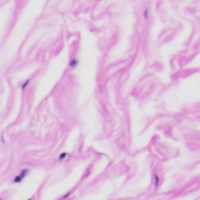

Supplement: S1 Database Files — (ZIP) [file pone.0141556.s001.zip › DATABASE/CLASS1TIF/zona44.tif]

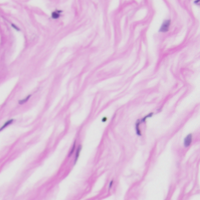

Supplement: S1 Database Files — (ZIP) [file pone.0141556.s001.zip › DATABASE/CLASS1TIF/zona45.tif]

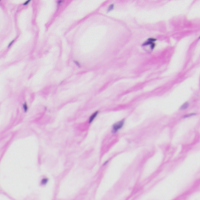

Supplement: S1 Database Files — (ZIP) [file pone.0141556.s001.zip › DATABASE/CLASS1TIF/zona46.tif]

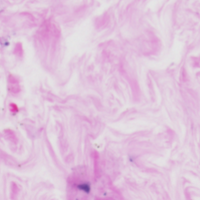

Supplement: S1 Database Files — (ZIP) [file pone.0141556.s001.zip › DATABASE/CLASS1TIF/zona47.tif]

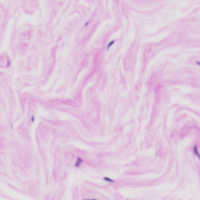

Supplement: S1 Database Files — (ZIP) [file pone.0141556.s001.zip › DATABASE/CLASS1TIF/zona48.tif]

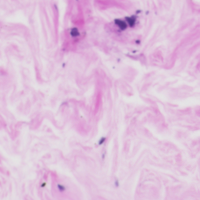

Supplement: S1 Database Files — (ZIP) [file pone.0141556.s001.zip › DATABASE/CLASS1TIF/zona49.tif]

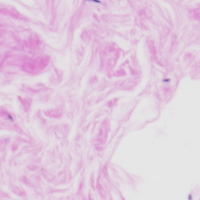

Supplement: S1 Database Files — (ZIP) [file pone.0141556.s001.zip › DATABASE/CLASS1TIF/zona50.tif]

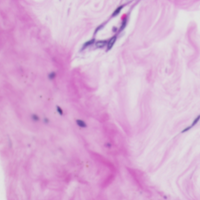

Supplement: S1 Database Files — (ZIP) [file pone.0141556.s001.zip › DATABASE/CLASS1TIF/zona51.tif]

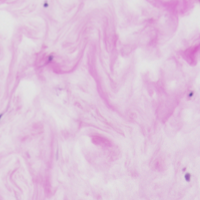

Supplement: S1 Database Files — (ZIP) [file pone.0141556.s001.zip › DATABASE/CLASS1TIF/zona52.tif]

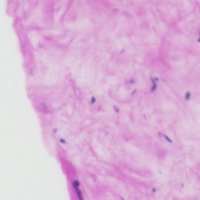

Supplement: S1 Database Files — (ZIP) [file pone.0141556.s001.zip › DATABASE/CLASS1TIF/zona53.tif]

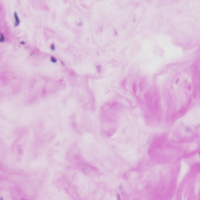

Supplement: S1 Database Files — (ZIP) [file pone.0141556.s001.zip › DATABASE/CLASS1TIF/zona54.tif]

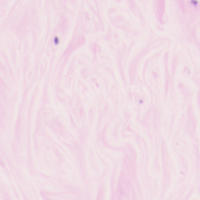

Supplement: S1 Database Files — (ZIP) [file pone.0141556.s001.zip › DATABASE/CLASS1TIF/zona55.tif]

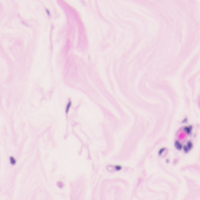

Supplement: S1 Database Files — (ZIP) [file pone.0141556.s001.zip › DATABASE/CLASS1TIF/zona56.tif]

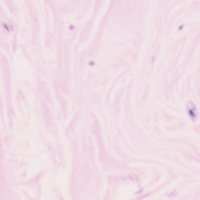

Supplement: S1 Database Files — (ZIP) [file pone.0141556.s001.zip › DATABASE/CLASS1TIF/zona57.tif]

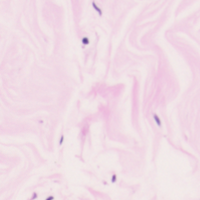

Supplement: S1 Database Files — (ZIP) [file pone.0141556.s001.zip › DATABASE/CLASS1TIF/zona58.tif]

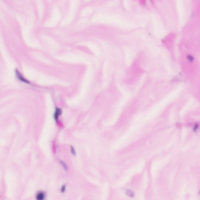

Supplement: S1 Database Files — (ZIP) [file pone.0141556.s001.zip › DATABASE/CLASS1TIF/zona59.tif]

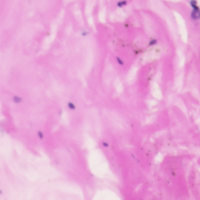

Supplement: S1 Database Files — (ZIP) [file pone.0141556.s001.zip › DATABASE/CLASS1TIF/zona60.tif]

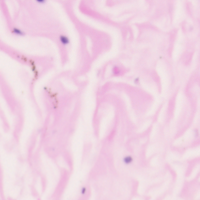

Supplement: S1 Database Files — (ZIP) [file pone.0141556.s001.zip › DATABASE/CLASS1TIF/zona61.tif]

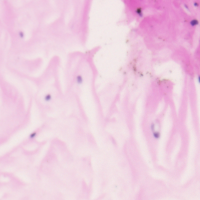

Supplement: S1 Database Files — (ZIP) [file pone.0141556.s001.zip › DATABASE/CLASS1TIF/zona62.tif]

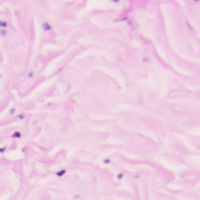

Supplement: S1 Database Files — (ZIP) [file pone.0141556.s001.zip › DATABASE/CLASS1TIF/zona63.tif]

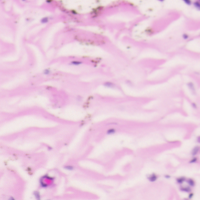

Supplement: S1 Database Files — (ZIP) [file pone.0141556.s001.zip › DATABASE/CLASS1TIF/zona64.tif]

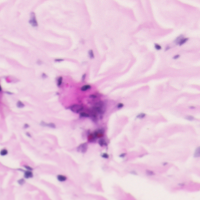

Supplement: S1 Database Files — (ZIP) [file pone.0141556.s001.zip › DATABASE/CLASS1TIF/zona65.tif]

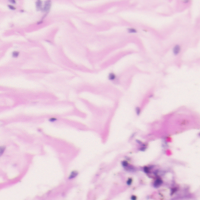

Supplement: S1 Database Files — (ZIP) [file pone.0141556.s001.zip › DATABASE/CLASS1TIF/zona66.tif]

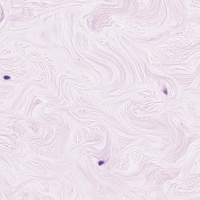

Supplement: S1 Database Files — (ZIP) [file pone.0141556.s001.zip › DATABASE/CLASS1TIF/zona67.tif]

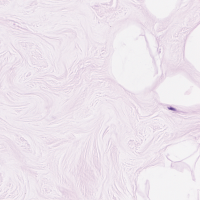

Supplement: S1 Database Files — (ZIP) [file pone.0141556.s001.zip › DATABASE/CLASS1TIF/zona68.tif]

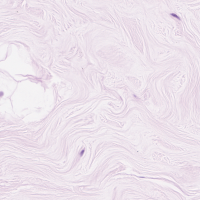

Supplement: S1 Database Files — (ZIP) [file pone.0141556.s001.zip › DATABASE/CLASS1TIF/zona69.tif]

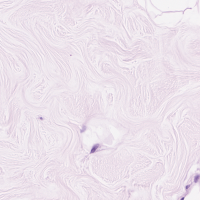

Supplement: S1 Database Files — (ZIP) [file pone.0141556.s001.zip › DATABASE/CLASS1TIF/zona70.tif]

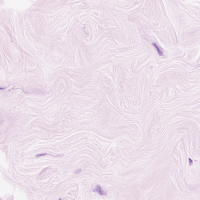

Supplement: S1 Database Files — (ZIP) [file pone.0141556.s001.zip › DATABASE/CLASS1TIF/zona71.tif]

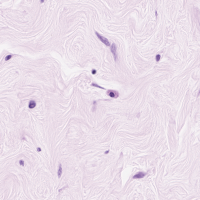

Supplement: S1 Database Files — (ZIP) [file pone.0141556.s001.zip › DATABASE/CLASS1TIF/zona72.tif]

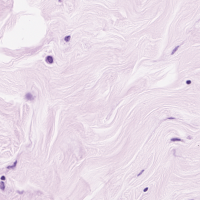

Supplement: S1 Database Files — (ZIP) [file pone.0141556.s001.zip › DATABASE/CLASS1TIF/zona73.tif]

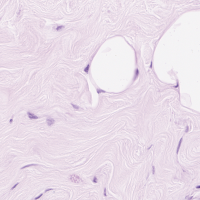

Supplement: S1 Database Files — (ZIP) [file pone.0141556.s001.zip › DATABASE/CLASS1TIF/zona74.tif]

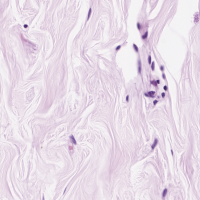

Supplement: S1 Database Files — (ZIP) [file pone.0141556.s001.zip › DATABASE/CLASS1TIF/zona75.tif]

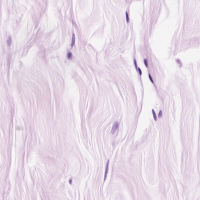

Supplement: S1 Database Files — (ZIP) [file pone.0141556.s001.zip › DATABASE/CLASS1TIF/zona76.tif]

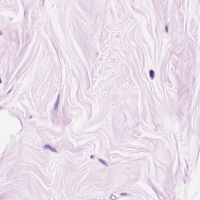

Supplement: S1 Database Files — (ZIP) [file pone.0141556.s001.zip › DATABASE/CLASS1TIF/zona77.tif]

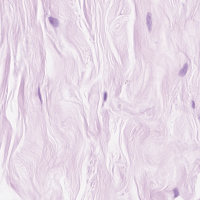

Supplement: S1 Database Files — (ZIP) [file pone.0141556.s001.zip › DATABASE/CLASS1TIF/zona78.tif]

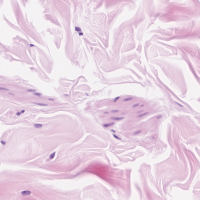

Supplement: S1 Database Files — (ZIP) [file pone.0141556.s001.zip › DATABASE/CLASS1TIF/zona79.tif]

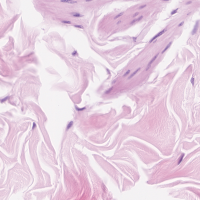

Supplement: S1 Database Files — (ZIP) [file pone.0141556.s001.zip › DATABASE/CLASS1TIF/zona80.tif]

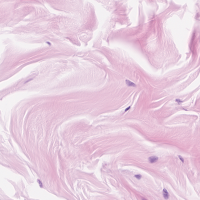

Supplement: S1 Database Files — (ZIP) [file pone.0141556.s001.zip › DATABASE/CLASS1TIF/zona81.tif]

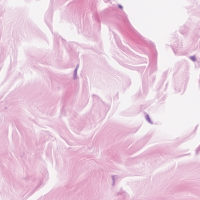

Supplement: S1 Database Files — (ZIP) [file pone.0141556.s001.zip › DATABASE/CLASS1TIF/zona82.tif]

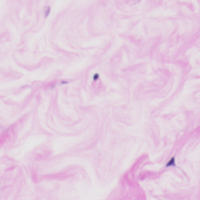

Supplement: S1 Database Files — (ZIP) [file pone.0141556.s001.zip › DATABASE/CLASS1TIF/zona83.tif]

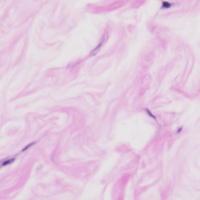

Supplement: S1 Database Files — (ZIP) [file pone.0141556.s001.zip › DATABASE/CLASS1TIF/zona84.tif]

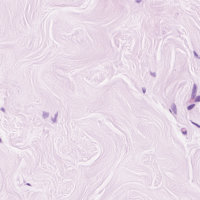

Supplement: S1 Database Files — (ZIP) [file pone.0141556.s001.zip › DATABASE/CLASS1TIF/zona85.tif]

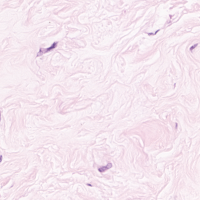

Supplement: S1 Database Files — (ZIP) [file pone.0141556.s001.zip › DATABASE/CLASS1TIF/zona86.tif]

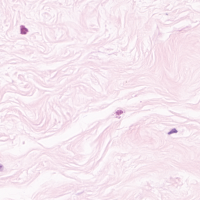

Supplement: S1 Database Files — (ZIP) [file pone.0141556.s001.zip › DATABASE/CLASS1TIF/zona87.tif]

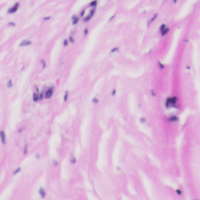

Supplement: S1 Database Files — (ZIP) [file pone.0141556.s001.zip › DATABASE/CLASS1TIF/zona90.tif]

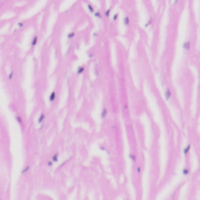

Supplement: S1 Database Files — (ZIP) [file pone.0141556.s001.zip › DATABASE/CLASS1TIF/zona91.tif]

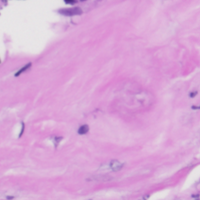

Supplement: S1 Database Files — (ZIP) [file pone.0141556.s001.zip › DATABASE/CLASS1TIF/zona92.tif]

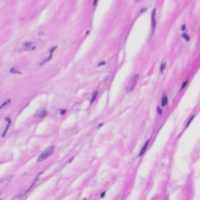

Supplement: S1 Database Files — (ZIP) [file pone.0141556.s001.zip › DATABASE/CLASS1TIF/zona93.tif]

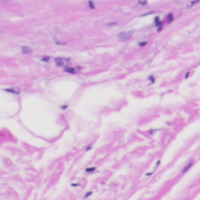

Supplement: S1 Database Files — (ZIP) [file pone.0141556.s001.zip › DATABASE/CLASS1TIF/zona94.tif]

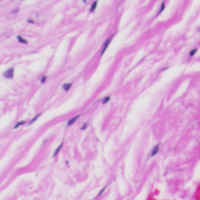

Supplement: S1 Database Files — (ZIP) [file pone.0141556.s001.zip › DATABASE/CLASS1TIF/zona95.tif]

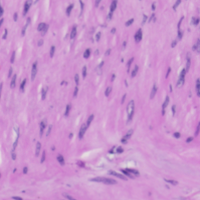

Supplement: S1 Database Files — (ZIP) [file pone.0141556.s001.zip › DATABASE/CLASS1TIF/zona96.tif]

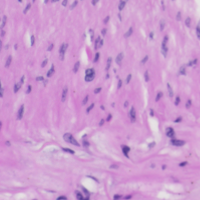

Supplement: S1 Database Files — (ZIP) [file pone.0141556.s001.zip › DATABASE/CLASS1TIF/zona97.tif]

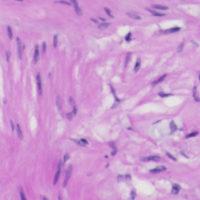

Supplement: S1 Database Files — (ZIP) [file pone.0141556.s001.zip › DATABASE/CLASS1TIF/zona98.tif]

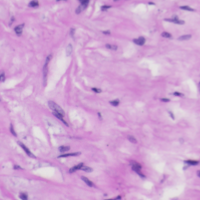

Supplement: S1 Database Files — (ZIP) [file pone.0141556.s001.zip › DATABASE/CLASS1TIF/zona99.tif]

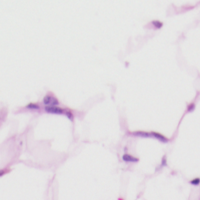

Supplement: S1 Database Files — (ZIP) [file pone.0141556.s001.zip › DATABASE/CLASS2TIF/zona1.tif]

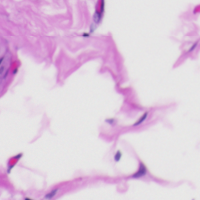

Supplement: S1 Database Files — (ZIP) [file pone.0141556.s001.zip › DATABASE/CLASS2TIF/zona10.tif]

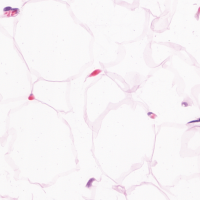

Supplement: S1 Database Files — (ZIP) [file pone.0141556.s001.zip › DATABASE/CLASS2TIF/zona100.tif]

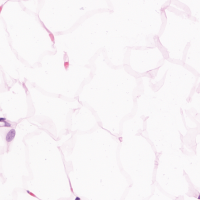

Supplement: S1 Database Files — (ZIP) [file pone.0141556.s001.zip › DATABASE/CLASS2TIF/zona101.tif]

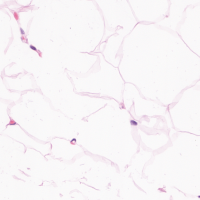

Supplement: S1 Database Files — (ZIP) [file pone.0141556.s001.zip › DATABASE/CLASS2TIF/zona102.tif]

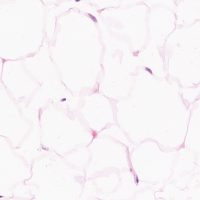

Supplement: S1 Database Files — (ZIP) [file pone.0141556.s001.zip › DATABASE/CLASS2TIF/zona103.tif]

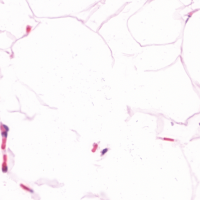

Supplement: S1 Database Files — (ZIP) [file pone.0141556.s001.zip › DATABASE/CLASS2TIF/zona104.tif]

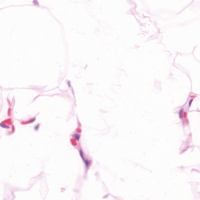

Supplement: S1 Database Files — (ZIP) [file pone.0141556.s001.zip › DATABASE/CLASS2TIF/zona105.tif]

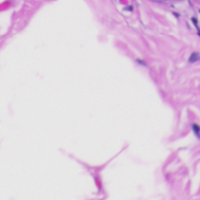

Supplement: S1 Database Files — (ZIP) [file pone.0141556.s001.zip › DATABASE/CLASS2TIF/zona11.tif]

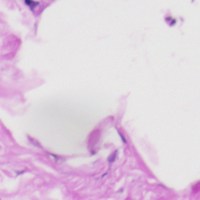

Supplement: S1 Database Files — (ZIP) [file pone.0141556.s001.zip › DATABASE/CLASS2TIF/zona12.tif]

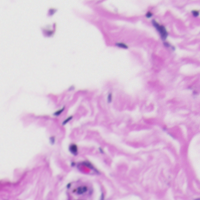

Supplement: S1 Database Files — (ZIP) [file pone.0141556.s001.zip › DATABASE/CLASS2TIF/zona13.tif]

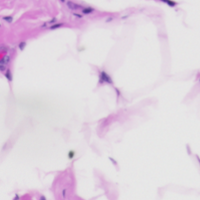

Supplement: S1 Database Files — (ZIP) [file pone.0141556.s001.zip › DATABASE/CLASS2TIF/zona14.tif]

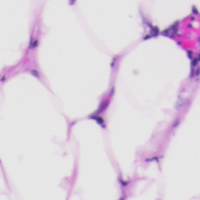

Supplement: S1 Database Files — (ZIP) [file pone.0141556.s001.zip › DATABASE/CLASS2TIF/zona15.tif]

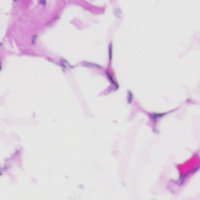

Supplement: S1 Database Files — (ZIP) [file pone.0141556.s001.zip › DATABASE/CLASS2TIF/zona16.tif]

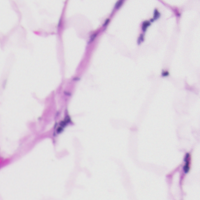

Supplement: S1 Database Files — (ZIP) [file pone.0141556.s001.zip › DATABASE/CLASS2TIF/zona17.tif]

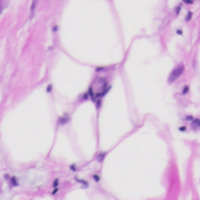

Supplement: S1 Database Files — (ZIP) [file pone.0141556.s001.zip › DATABASE/CLASS2TIF/zona18.tif]

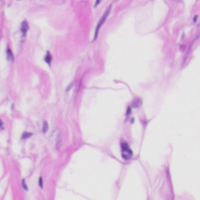

Supplement: S1 Database Files — (ZIP) [file pone.0141556.s001.zip › DATABASE/CLASS2TIF/zona19.tif]

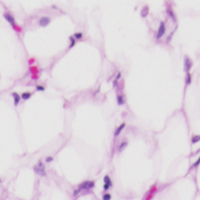

Supplement: S1 Database Files — (ZIP) [file pone.0141556.s001.zip › DATABASE/CLASS2TIF/zona2.tif]

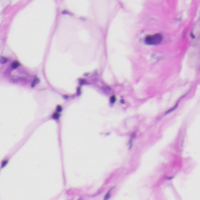

Supplement: S1 Database Files — (ZIP) [file pone.0141556.s001.zip › DATABASE/CLASS2TIF/zona20.tif]

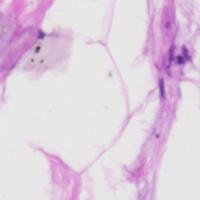

Supplement: S1 Database Files — (ZIP) [file pone.0141556.s001.zip › DATABASE/CLASS2TIF/zona21.tif]

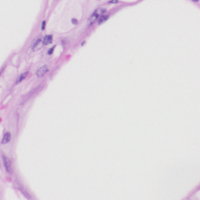

Supplement: S1 Database Files — (ZIP) [file pone.0141556.s001.zip › DATABASE/CLASS2TIF/zona22.tif]

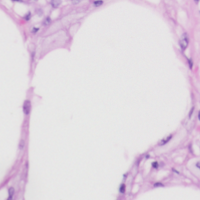

Supplement: S1 Database Files — (ZIP) [file pone.0141556.s001.zip › DATABASE/CLASS2TIF/zona23.tif]

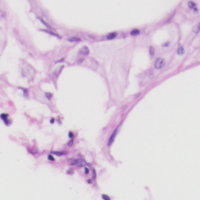

Supplement: S1 Database Files — (ZIP) [file pone.0141556.s001.zip › DATABASE/CLASS2TIF/zona24.tif]

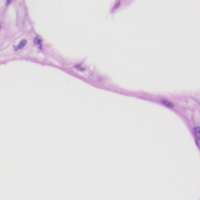

Supplement: S1 Database Files — (ZIP) [file pone.0141556.s001.zip › DATABASE/CLASS2TIF/zona25.tif]

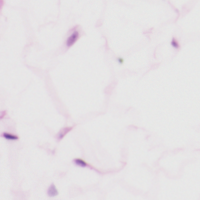

Supplement: S1 Database Files — (ZIP) [file pone.0141556.s001.zip › DATABASE/CLASS2TIF/zona26.tif]

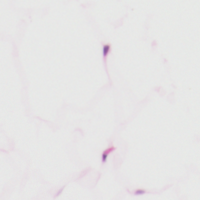

Supplement: S1 Database Files — (ZIP) [file pone.0141556.s001.zip › DATABASE/CLASS2TIF/zona27.tif]

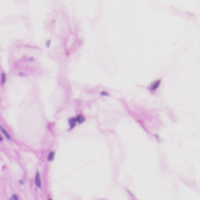

Supplement: S1 Database Files — (ZIP) [file pone.0141556.s001.zip › DATABASE/CLASS2TIF/zona28.tif]

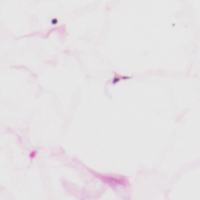

Supplement: S1 Database Files — (ZIP) [file pone.0141556.s001.zip › DATABASE/CLASS2TIF/zona29.tif]

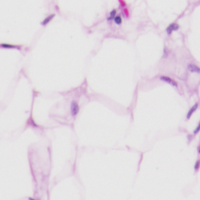

Supplement: S1 Database Files — (ZIP) [file pone.0141556.s001.zip › DATABASE/CLASS2TIF/zona3.tif]

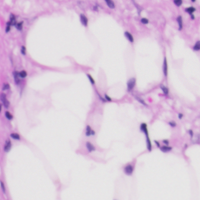

Supplement: S1 Database Files — (ZIP) [file pone.0141556.s001.zip › DATABASE/CLASS2TIF/zona30.tif]
