# Supplementary figures and images for: Influence of Texture and Colour in Breast TMA Classification (part 1 of 2)
Source: PLoS One. 2015 Oct 29;10(10):e0141556. doi: 10.1371/journal.pone.0141556 (PMC4626403; doi:10.1371/journal.pone.0141556)

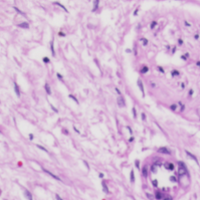

Supplement: S1 Database Files — (ZIP) [file pone.0141556.s001.zip › DATABASE/CLASS1TIF/zona100.tif]

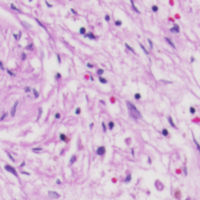

Supplement: S1 Database Files — (ZIP) [file pone.0141556.s001.zip › DATABASE/CLASS1TIF/zona101.tif]

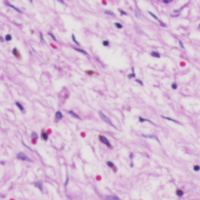

Supplement: S1 Database Files — (ZIP) [file pone.0141556.s001.zip › DATABASE/CLASS1TIF/zona102.tif]

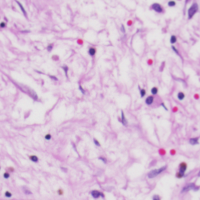

Supplement: S1 Database Files — (ZIP) [file pone.0141556.s001.zip › DATABASE/CLASS1TIF/zona103.tif]

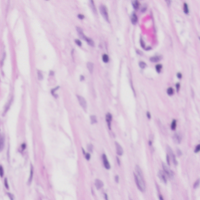

Supplement: S1 Database Files — (ZIP) [file pone.0141556.s001.zip › DATABASE/CLASS1TIF/zona106.tif]

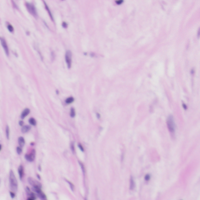

Supplement: S1 Database Files — (ZIP) [file pone.0141556.s001.zip › DATABASE/CLASS1TIF/zona107.tif]

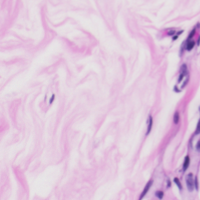

Supplement: S1 Database Files — (ZIP) [file pone.0141556.s001.zip › DATABASE/CLASS1TIF/zona108.tif]

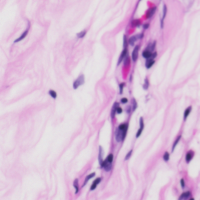

Supplement: S1 Database Files — (ZIP) [file pone.0141556.s001.zip › DATABASE/CLASS1TIF/zona109.tif]

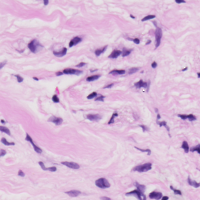

Supplement: S1 Database Files — (ZIP) [file pone.0141556.s001.zip › DATABASE/CLASS1TIF/zona110.tif]

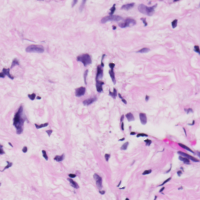

Supplement: S1 Database Files — (ZIP) [file pone.0141556.s001.zip › DATABASE/CLASS1TIF/zona111.tif]

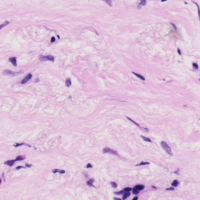

Supplement: S1 Database Files — (ZIP) [file pone.0141556.s001.zip › DATABASE/CLASS1TIF/zona112.tif]

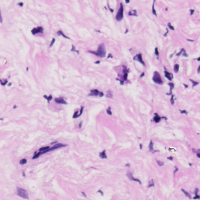

Supplement: S1 Database Files — (ZIP) [file pone.0141556.s001.zip › DATABASE/CLASS1TIF/zona113.tif]

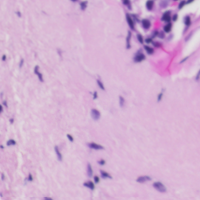

Supplement: S1 Database Files — (ZIP) [file pone.0141556.s001.zip › DATABASE/CLASS1TIF/zona114.tif]

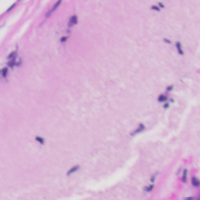

Supplement: S1 Database Files — (ZIP) [file pone.0141556.s001.zip › DATABASE/CLASS1TIF/zona115.tif]

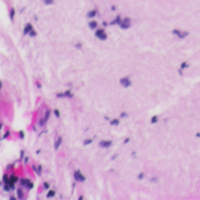

Supplement: S1 Database Files — (ZIP) [file pone.0141556.s001.zip › DATABASE/CLASS1TIF/zona116.tif]

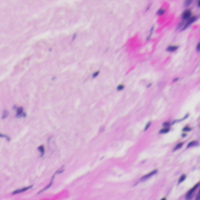

Supplement: S1 Database Files — (ZIP) [file pone.0141556.s001.zip › DATABASE/CLASS1TIF/zona117.tif]

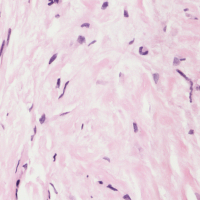

Supplement: S1 Database Files — (ZIP) [file pone.0141556.s001.zip › DATABASE/CLASS1TIF/zona118.tif]

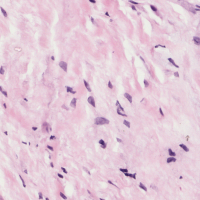

Supplement: S1 Database Files — (ZIP) [file pone.0141556.s001.zip › DATABASE/CLASS1TIF/zona119.tif]

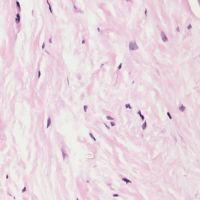

Supplement: S1 Database Files — (ZIP) [file pone.0141556.s001.zip › DATABASE/CLASS1TIF/zona120.tif]

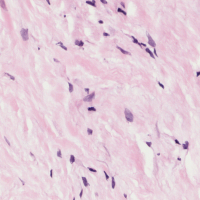

Supplement: S1 Database Files — (ZIP) [file pone.0141556.s001.zip › DATABASE/CLASS1TIF/zona121.tif]

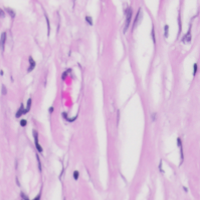

Supplement: S1 Database Files — (ZIP) [file pone.0141556.s001.zip › DATABASE/CLASS1TIF/zona122.tif]

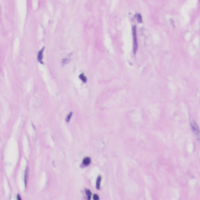

Supplement: S1 Database Files — (ZIP) [file pone.0141556.s001.zip › DATABASE/CLASS1TIF/zona123.tif]

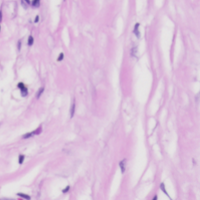

Supplement: S1 Database Files — (ZIP) [file pone.0141556.s001.zip › DATABASE/CLASS1TIF/zona124.tif]

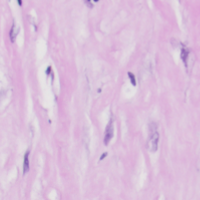

Supplement: S1 Database Files — (ZIP) [file pone.0141556.s001.zip › DATABASE/CLASS1TIF/zona125.tif]

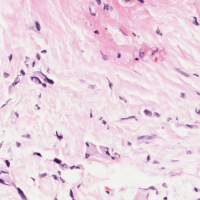

Supplement: S1 Database Files — (ZIP) [file pone.0141556.s001.zip › DATABASE/CLASS1TIF/zona126.tif]

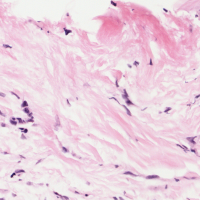

Supplement: S1 Database Files — (ZIP) [file pone.0141556.s001.zip › DATABASE/CLASS1TIF/zona127.tif]

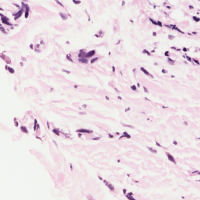

Supplement: S1 Database Files — (ZIP) [file pone.0141556.s001.zip › DATABASE/CLASS1TIF/zona128.tif]

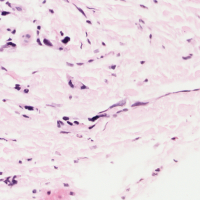

Supplement: S1 Database Files — (ZIP) [file pone.0141556.s001.zip › DATABASE/CLASS1TIF/zona129.tif]

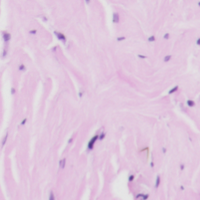

Supplement: S1 Database Files — (ZIP) [file pone.0141556.s001.zip › DATABASE/CLASS1TIF/zona130.tif]

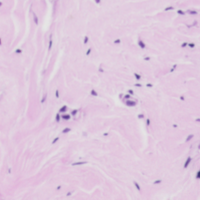

Supplement: S1 Database Files — (ZIP) [file pone.0141556.s001.zip › DATABASE/CLASS1TIF/zona131.tif]

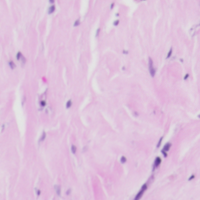

Supplement: S1 Database Files — (ZIP) [file pone.0141556.s001.zip › DATABASE/CLASS1TIF/zona132.tif]

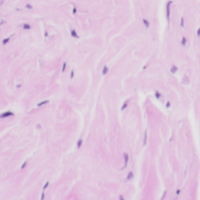

Supplement: S1 Database Files — (ZIP) [file pone.0141556.s001.zip › DATABASE/CLASS1TIF/zona133.tif]

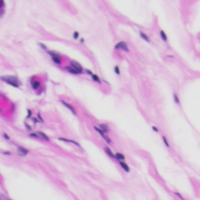

Supplement: S1 Database Files — (ZIP) [file pone.0141556.s001.zip › DATABASE/CLASS1TIF/zona134.tif]

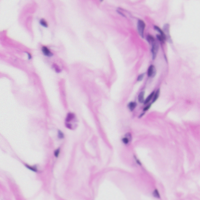

Supplement: S1 Database Files — (ZIP) [file pone.0141556.s001.zip › DATABASE/CLASS1TIF/zona135.tif]

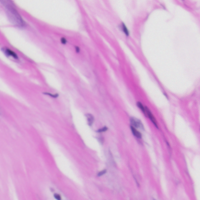

Supplement: S1 Database Files — (ZIP) [file pone.0141556.s001.zip › DATABASE/CLASS1TIF/zona136.tif]

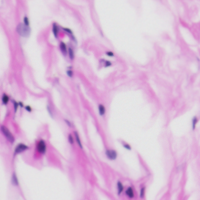

Supplement: S1 Database Files — (ZIP) [file pone.0141556.s001.zip › DATABASE/CLASS1TIF/zona137.tif]

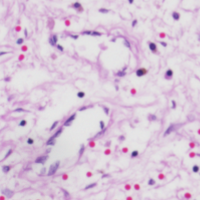

Supplement: S1 Database Files — (ZIP) [file pone.0141556.s001.zip › DATABASE/CLASS1TIF/zona138.tif]

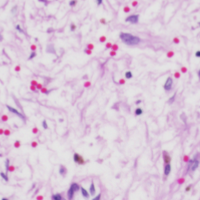

Supplement: S1 Database Files — (ZIP) [file pone.0141556.s001.zip › DATABASE/CLASS1TIF/zona139.tif]

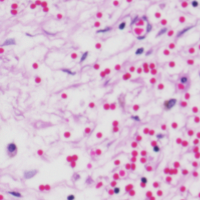

Supplement: S1 Database Files — (ZIP) [file pone.0141556.s001.zip › DATABASE/CLASS1TIF/zona140.tif]

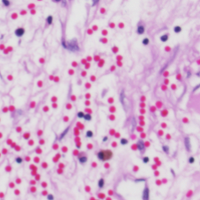

Supplement: S1 Database Files — (ZIP) [file pone.0141556.s001.zip › DATABASE/CLASS1TIF/zona141.tif]

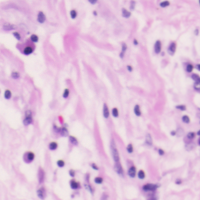

Supplement: S1 Database Files — (ZIP) [file pone.0141556.s001.zip › DATABASE/CLASS1TIF/zona142.tif]

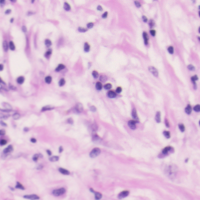

Supplement: S1 Database Files — (ZIP) [file pone.0141556.s001.zip › DATABASE/CLASS1TIF/zona143.tif]

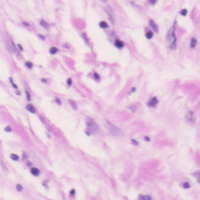

Supplement: S1 Database Files — (ZIP) [file pone.0141556.s001.zip › DATABASE/CLASS1TIF/zona144.tif]

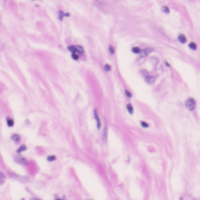

Supplement: S1 Database Files — (ZIP) [file pone.0141556.s001.zip › DATABASE/CLASS1TIF/zona145.tif]

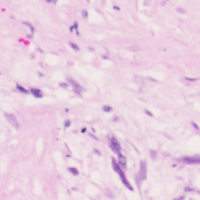

Supplement: S1 Database Files — (ZIP) [file pone.0141556.s001.zip › DATABASE/CLASS1TIF/zona146.tif]

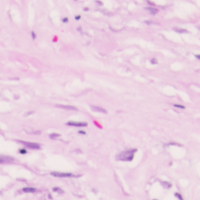

Supplement: S1 Database Files — (ZIP) [file pone.0141556.s001.zip › DATABASE/CLASS1TIF/zona147.tif]

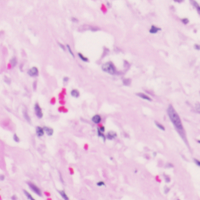

Supplement: S1 Database Files — (ZIP) [file pone.0141556.s001.zip › DATABASE/CLASS1TIF/zona148.tif]

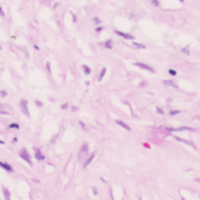

Supplement: S1 Database Files — (ZIP) [file pone.0141556.s001.zip › DATABASE/CLASS1TIF/zona149.tif]

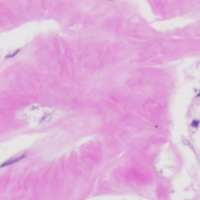

Supplement: S1 Database Files — (ZIP) [file pone.0141556.s001.zip › DATABASE/CLASS1TIF/zona15.tif]

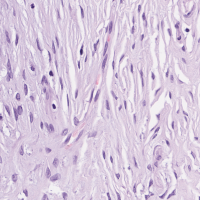

Supplement: S1 Database Files — (ZIP) [file pone.0141556.s001.zip › DATABASE/CLASS1TIF/zona150.tif]

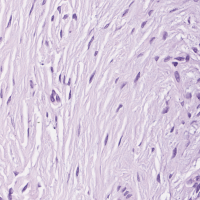

Supplement: S1 Database Files — (ZIP) [file pone.0141556.s001.zip › DATABASE/CLASS1TIF/zona151.tif]

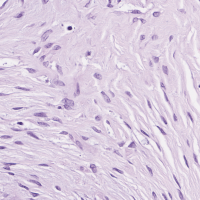

Supplement: S1 Database Files — (ZIP) [file pone.0141556.s001.zip › DATABASE/CLASS1TIF/zona152.tif]

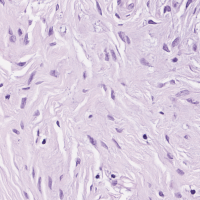

Supplement: S1 Database Files — (ZIP) [file pone.0141556.s001.zip › DATABASE/CLASS1TIF/zona153.tif]

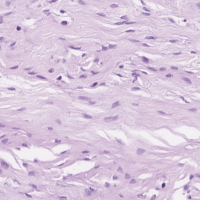

Supplement: S1 Database Files — (ZIP) [file pone.0141556.s001.zip › DATABASE/CLASS1TIF/zona154.tif]

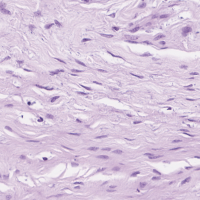

Supplement: S1 Database Files — (ZIP) [file pone.0141556.s001.zip › DATABASE/CLASS1TIF/zona155.tif]

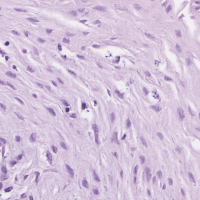

Supplement: S1 Database Files — (ZIP) [file pone.0141556.s001.zip › DATABASE/CLASS1TIF/zona156.tif]

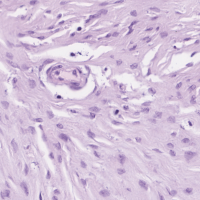

Supplement: S1 Database Files — (ZIP) [file pone.0141556.s001.zip › DATABASE/CLASS1TIF/zona157.tif]

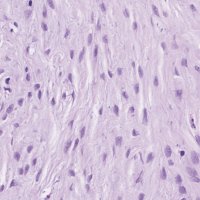

Supplement: S1 Database Files — (ZIP) [file pone.0141556.s001.zip › DATABASE/CLASS1TIF/zona158.tif]

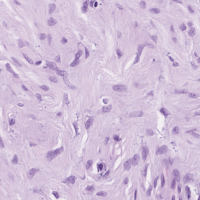

Supplement: S1 Database Files — (ZIP) [file pone.0141556.s001.zip › DATABASE/CLASS1TIF/zona159.tif]

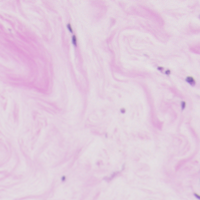

Supplement: S1 Database Files — (ZIP) [file pone.0141556.s001.zip › DATABASE/CLASS1TIF/zona16.tif]

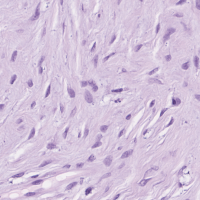

Supplement: S1 Database Files — (ZIP) [file pone.0141556.s001.zip › DATABASE/CLASS1TIF/zona160.tif]

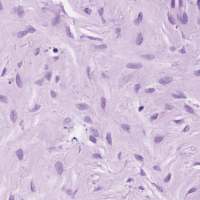

Supplement: S1 Database Files — (ZIP) [file pone.0141556.s001.zip › DATABASE/CLASS1TIF/zona161.tif]

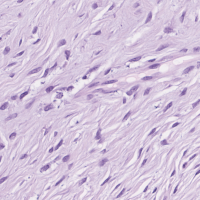

Supplement: S1 Database Files — (ZIP) [file pone.0141556.s001.zip › DATABASE/CLASS1TIF/zona162.tif]

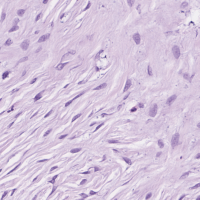

Supplement: S1 Database Files — (ZIP) [file pone.0141556.s001.zip › DATABASE/CLASS1TIF/zona163.tif]

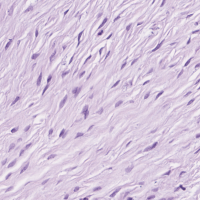

Supplement: S1 Database Files — (ZIP) [file pone.0141556.s001.zip › DATABASE/CLASS1TIF/zona164.tif]

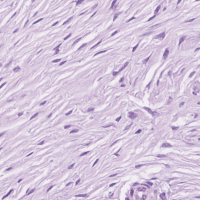

Supplement: S1 Database Files — (ZIP) [file pone.0141556.s001.zip › DATABASE/CLASS1TIF/zona165.tif]

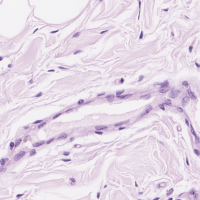

Supplement: S1 Database Files — (ZIP) [file pone.0141556.s001.zip › DATABASE/CLASS1TIF/zona166.tif]

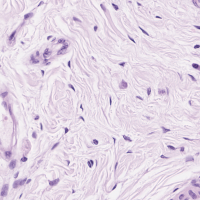

Supplement: S1 Database Files — (ZIP) [file pone.0141556.s001.zip › DATABASE/CLASS1TIF/zona167.tif]

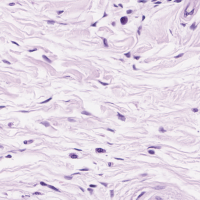

Supplement: S1 Database Files — (ZIP) [file pone.0141556.s001.zip › DATABASE/CLASS1TIF/zona168.tif]

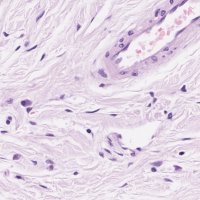

Supplement: S1 Database Files — (ZIP) [file pone.0141556.s001.zip › DATABASE/CLASS1TIF/zona169.tif]

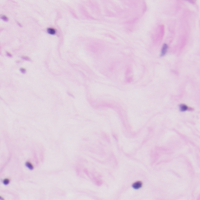

Supplement: S1 Database Files — (ZIP) [file pone.0141556.s001.zip › DATABASE/CLASS1TIF/zona17.tif]

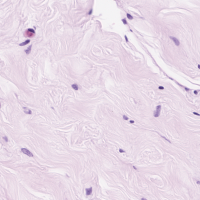

Supplement: S1 Database Files — (ZIP) [file pone.0141556.s001.zip › DATABASE/CLASS1TIF/zona170.tif]

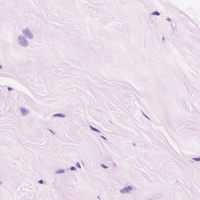

Supplement: S1 Database Files — (ZIP) [file pone.0141556.s001.zip › DATABASE/CLASS1TIF/zona171.tif]

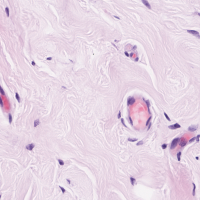

Supplement: S1 Database Files — (ZIP) [file pone.0141556.s001.zip › DATABASE/CLASS1TIF/zona172.tif]

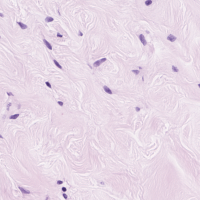

Supplement: S1 Database Files — (ZIP) [file pone.0141556.s001.zip › DATABASE/CLASS1TIF/zona173.tif]

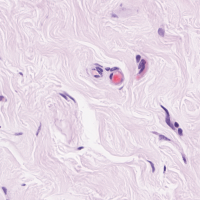

Supplement: S1 Database Files — (ZIP) [file pone.0141556.s001.zip › DATABASE/CLASS1TIF/zona174.tif]

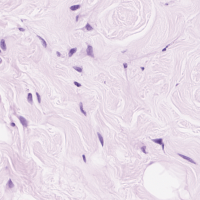

Supplement: S1 Database Files — (ZIP) [file pone.0141556.s001.zip › DATABASE/CLASS1TIF/zona175.tif]

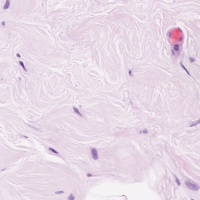

Supplement: S1 Database Files — (ZIP) [file pone.0141556.s001.zip › DATABASE/CLASS1TIF/zona176.tif]

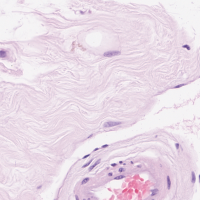

Supplement: S1 Database Files — (ZIP) [file pone.0141556.s001.zip › DATABASE/CLASS1TIF/zona177.tif]

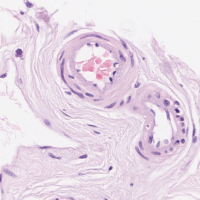

Supplement: S1 Database Files — (ZIP) [file pone.0141556.s001.zip › DATABASE/CLASS1TIF/zona178.tif]

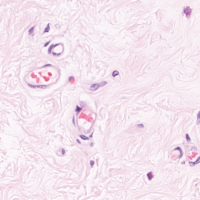

Supplement: S1 Database Files — (ZIP) [file pone.0141556.s001.zip › DATABASE/CLASS1TIF/zona179.tif]

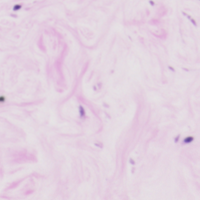

Supplement: S1 Database Files — (ZIP) [file pone.0141556.s001.zip › DATABASE/CLASS1TIF/zona18.tif]

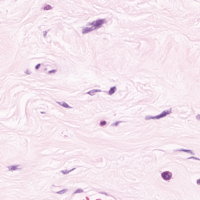

Supplement: S1 Database Files — (ZIP) [file pone.0141556.s001.zip › DATABASE/CLASS1TIF/zona180.tif]

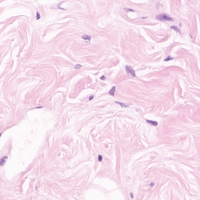

Supplement: S1 Database Files — (ZIP) [file pone.0141556.s001.zip › DATABASE/CLASS1TIF/zona181.tif]

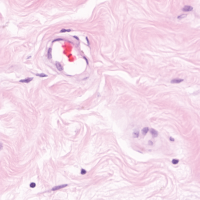

Supplement: S1 Database Files — (ZIP) [file pone.0141556.s001.zip › DATABASE/CLASS1TIF/zona182.tif]

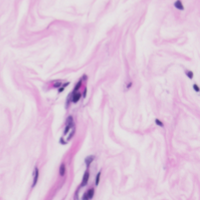

Supplement: S1 Database Files — (ZIP) [file pone.0141556.s001.zip › DATABASE/CLASS1TIF/zona183.tif]

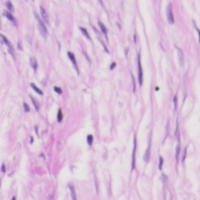

Supplement: S1 Database Files — (ZIP) [file pone.0141556.s001.zip › DATABASE/CLASS1TIF/zona184.tif]

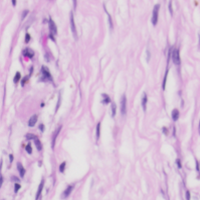

Supplement: S1 Database Files — (ZIP) [file pone.0141556.s001.zip › DATABASE/CLASS1TIF/zona185.tif]

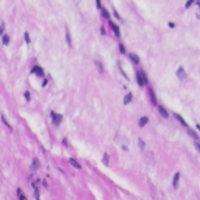

Supplement: S1 Database Files — (ZIP) [file pone.0141556.s001.zip › DATABASE/CLASS1TIF/zona186.tif]

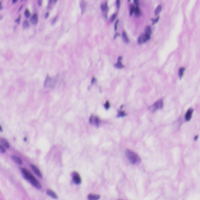

Supplement: S1 Database Files — (ZIP) [file pone.0141556.s001.zip › DATABASE/CLASS1TIF/zona187.tif]

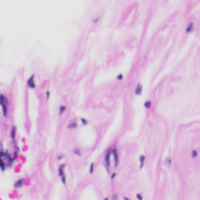

Supplement: S1 Database Files — (ZIP) [file pone.0141556.s001.zip › DATABASE/CLASS1TIF/zona188.tif]

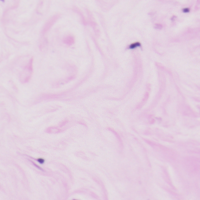

Supplement: S1 Database Files — (ZIP) [file pone.0141556.s001.zip › DATABASE/CLASS1TIF/zona19.tif]

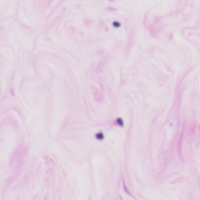

Supplement: S1 Database Files — (ZIP) [file pone.0141556.s001.zip › DATABASE/CLASS1TIF/zona20.tif]

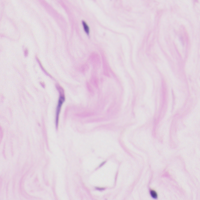

Supplement: S1 Database Files — (ZIP) [file pone.0141556.s001.zip › DATABASE/CLASS1TIF/zona21.tif]

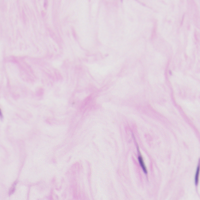

Supplement: S1 Database Files — (ZIP) [file pone.0141556.s001.zip › DATABASE/CLASS1TIF/zona22.tif]

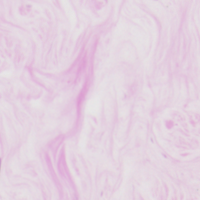

Supplement: S1 Database Files — (ZIP) [file pone.0141556.s001.zip › DATABASE/CLASS1TIF/zona23.tif]

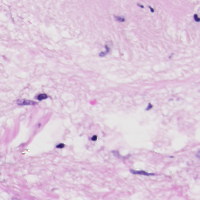

Supplement: S1 Database Files — (ZIP) [file pone.0141556.s001.zip › DATABASE/CLASS1TIF/zona24.tif]

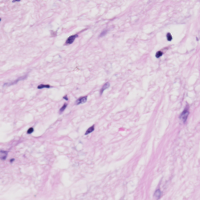

Supplement: S1 Database Files — (ZIP) [file pone.0141556.s001.zip › DATABASE/CLASS1TIF/zona25.tif]

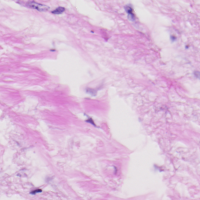

Supplement: S1 Database Files — (ZIP) [file pone.0141556.s001.zip › DATABASE/CLASS1TIF/zona26.tif]

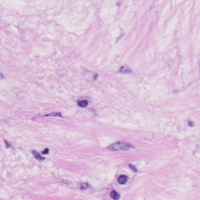

Supplement: S1 Database Files — (ZIP) [file pone.0141556.s001.zip › DATABASE/CLASS1TIF/zona27.tif]
